# mRNA Engineering for the Efficient Chaperone-Mediated Co-Translational Folding of Recombinant Proteins in *Escherichia coli*

**DOI:** 10.3390/ijms20133163

**Published:** 2019-06-28

**Authors:** Le Minh Bui, Almando Geraldi, Thi Thuy Nguyen, Jun Hyoung Lee, Ju Young Lee, Byung-Kwan Cho, Sun Chang Kim

**Affiliations:** 1 KAIST Institute for BioCentury, Korea Advanced Institute of Science and Technology (KAIST), Daejeon 34141, Korea; 2 NTT Hi-Tech Institute, Nguyen Tat Thanh University (NTTU), Ho Chi Minh City 700000, Vietnam; 3 Department of Biological Sciences, Korea Advanced Institute of Science and Technology (KAIST), Daejeon 34141, Korea; 4 Biology Department, Science and Technology Faculty, Universitas Airlangga Mulyorejo, Surabaya 60115, Indonesia; 5 Center for Bio-based Chemistry, Korea Research Institute of Chemical Technology (KRICT), Ulsan 44429, Korea; 6 Intelligent Synthetic Biology Center, Korea Advanced Institute of Science and Technology (KAIST), Daejeon 34141, Korea

**Keywords:** molecular chaperone, DnaJK-GrpE, co-translational folding, RNA scaffold, two-cistron expression, inclusion body, recombinant protein, protein aggregation, mRNA engineering, protein solubilization

## Abstract

The production of soluble, functional recombinant proteins by engineered bacterial hosts is challenging. Natural molecular chaperone systems have been used to solubilize various recombinant proteins with limited success. Here, we attempted to facilitate chaperone-mediated folding by directing the molecular chaperones to their protein substrates before the co-translational folding process completed. To achieve this, we either anchored the bacterial chaperone DnaJ to the 3ʹ untranslated region of a target mRNA by fusing with an RNA-binding domain in the chaperone-recruiting mRNA scaffold (CRAS) system, or coupled the expression of DnaJ and a target recombinant protein using the overlapping stop-start codons 5ʹ-TAATG-3ʹ between the two genes in a chaperone-substrate co-localized expression (CLEX) system. By engineering the untranslated and intergenic sequences of the mRNA transcript, bacterial molecular chaperones are spatially constrained to the location of protein translation, expressing selected aggregation-prone proteins in their functionally active, soluble form. Our mRNA engineering methods surpassed the in-vivo solubilization efficiency of the simple DnaJ chaperone co-overexpression method, thus providing more effective tools for producing soluble therapeutic proteins and enzymes.

## 1. Introduction

Functional expression of recombinant proteins in prokaryotic hosts is of considerable industrial interest, with applications ranging from therapeutic proteins and recombinant enzyme production to metabolic engineering for synthesizing value-added biomolecules [1,2]. However, recombinant protein overexpression in prokaryotic hosts commonly results in protein aggregate formations, termed inclusion bodies [3], which far exceed the host folding capacity. Accordingly, costly and time-consuming in-vitro refolding processes have been widely applied to recover functionally active proteins in their soluble forms. Furthermore, inclusion body formation is generally harmful to the host through increased cellular metabolic burden and intermolecular interactions with other cellular components [4,5], reducing cell viability and overall production yield. 

*In vivo* and *in vitro* strategies to solubilize recombinant proteins to an active conformation during the initial production process [6] include solubility-enhancing tags to intrinsically prevent misfolded protein formation in-vivo [7,8,9], physicochemical condition modification to balance translational and folding processes [10,11], and molecular chaperone overexpression to improve host disaggregation and folding capacity [12,13,14]. Molecular chaperones, which can assist cells in coping with stress-induced protein denaturation [15,16], constitute powerful tools to produce functionally active and water-soluble recombinant proteins. Among the options, the use of molecular chaperones is more cost-effective and less time-consuming as no post-translational processing and compromises in cell growth conditions are required. However, straightforward native chaperone co-overexpression with target proteins yields only limited solubilization during recombinant protein production. Although abundant molecular chaperones may be available through the chaperone co-expression approaches, these may not effectively diffuse through the crowded cytosol to interact in a timely fashion with a nascent polypeptide before the mis-foldings happen [17]. The rapid kinetics of the folding process that occurs during the translation of recombinant proteins easily results in the irreversible misfolding, aggregation, and degradation if they are not assisted by folding modulators in a timely fashion. Therefore, our strategy was to force molecular chaperone and nascent or translating peptide interaction to prevent further misfolding and aggregation, as well as to facilitate chaperone-mediated refolding processes. 

Accordingly, we transformed the 3′ untranslated region (3′UTR) of the mRNA encoding a target recombinant protein into a scaffold to anchor a molecular chaperone in proximity to the translational stop site. By such an arrangement, the protein translation is coupled with the refolding process. Alternatively, we placed molecular chaperone and target protein genes in tandem or in an overlapping arrangement to couple their translations, allowing the two proteins to interact rapidly during the translation process. The former approach, termed chaperone-recruiting mRNA scaffold (CRAS), was designed to prevent the aggregation and further misfolding of newly synthesized proteins prior to the final native folding. The latter approach, namely chaperone-substrate co-localized expression (CLEX), enabled protein involvement in the other’s translation and facilitated co-translational folding. We chose the chaperone and co-chaperones of the DnaK (Hsp70) chaperone system as the folding modulators employed in our designs, as they play a central role in the chaperone network, recognize a wide range of substrates, and directly work with the folding of nascent polypeptides [18,19]. In a typical DnaK functional cycle, the co-chaperone DnaJ (Hsp40) binds to the hydrophobic patches of unfolded polypeptides, transfers the substrate molecules to DnaK for the folding process. The folded polypeptides are released from DnaK by the nucleotide exchange factor GrpE and may be delivered to other chaperones for further foldings [20]. As the holdase that firstly interacts with unfolded polypeptides and prevent the further misfolding and aggregation, DnaJ also can function in a DnaK-independent manner [21,22]. Thus, we selected DnaJ as the co-chaperone targeted to the 3′UTR in CRAS system and to be co-overexpressed with a target protein in the CLEX system. For introducing an RNA binding function to DnaJ, we fused DnaJ to a human Nova-1 KH3 domain, which specifically binds toward a tetranucleotide UCAU motif in a single-stranded hairpin loop of an RNA molecule [23].

By engineering the mRNA encoding a target aggregation-prone recombinant protein with either an additional 3′UTR hairpin structure or the second cistron expressing DnaJ, we were able to outperform the simple DnaJ co-overexpression with target proteins in terms of producing soluble proteins. A target protein can be produced up to 90% in soluble fraction when the translation is assisted with the high local concentration of chaperones in CRAS/CLEX. Our systems can produce soluble target proteins even when the chaperone is unable to solubilize any appreciable amount of protein with the DnaJ co-overexpression. The combinations of various factors involved in the refolding/disaggregation mechanisms, such as the gene distances, number of hairpins, chaperone components, etc. of the two systems were examined to maximize the efficiency of the method.

## 2. Results

### 2.1. The CRAS System Enhances Post-Translational Refolding of Highly Insoluble Recombinant Proteins

To test our hypothesis that spatially tethering a molecular chaperone with a target protein facilitates correct protein folding, we utilized the *Escherichia coli* DnaK chaperone system for in vivo solubilization of structurally diverse and aggregation-prone proteins. The proteins of interest (POIs) were chosen with a large diversity, including human antibody (anti-Ras single chain variable fragment, ScFv [24]), its fusion form for targeted-drug delivery (ScFv fused to a antimicrobial peptide BR2, BR2-ScFv [25]), therapeutic protein (bone morphogenetic protein 2, BMP2 [26], leptin [27,28]), viral protease (HIV-1 protease, HIV1-Pr [29]), and metabolic enzymes from *E. coli* and yeast (UGD [30], UbiC [31], Adh1p [32]). These POIs have been reported to show high aggregation tendency when expressed in bacteria [25,33,34,35,36,37] or observed in our experiments.

**Figure 1:**
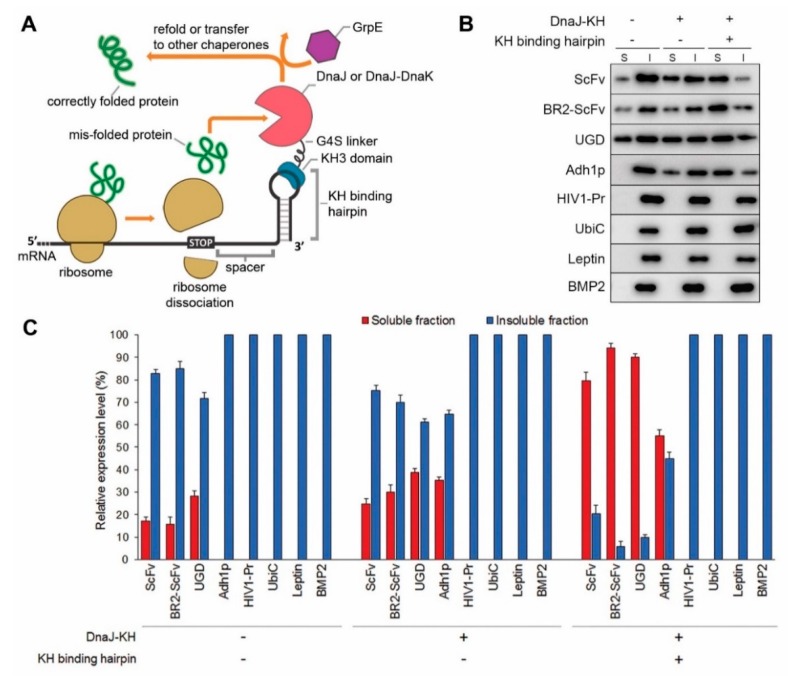
Solubilization of recombinant proteins by the chaperone-recruiting mRNA scaffold (CRAS) system. (**A**) Schematic representation of the CRAS system-mediated *in vivo* protein refolding process. A molecular chaperone (DnaJ, or DnaJ and DnaK fusion (DnaJK)) fused with the Nova-1 KH3 RNA binding domain was co-expressed with a target recombinant protein containing a KH cognate stem-loop sequence. KH-binding structure introduction into the mRNA 3′UTR allows fusion chaperone protein recruitment to the 3′UTR, facilitating nascent protein binding to prevent aggregation or for transfer to other molecular chaperones for proper folding. In the DnaK chaperone system, DnaJ is the first co-chaperone to bind to the substrate, DnaK is the main foldase responsible for the substrate re-folding, and GrpE as the nucleotide exchange factor releases the substrate from DnaK via an ATP-independent manner. The illustrated GrpE is either expressed from *E. coli* genome or overexpressed from a plasmid. (**B**) Western Blot analysis showing the soluble (S) and insoluble (I) fractions of recombinant proteins expressed using anti-His-tag antibody. The presence (+) and absence (−) of CRAS components, chaperone fusion DnaJ-KH, and the KH binding hairpin on mRNA 3′UTR are indicated. The cropped blots are shown with the black lines surrounding blots indicate the cropping lines. Full-length blots of these cropped images are presented in Supplementary Figure S2. (**C**) Solubilization of 8 selected aggregation-prone recombinant proteins (ScFv, HIV1-Pr, BR2-ScFv, UGD, Adh1p, UbiC, Leptin, and BMP2) upon co-expression with DnaJ-KH, or both DnaJ-KH and the 3′UTR hairpin loop. Soluble protein quantification was conducted using ImageJ v1.48 software (National Institutes of Health) on Coomassie Blue-stained SDS-PAGE images. Error bars indicate ± s. d. from three independent experiments.

DnaJ, the first co-chaperone of DnaK system to interact with the nascent proteins, binds to its substrate proteins and facilitates their interaction with foldase chaperones (e.g., DnaK or GroESL) to produce correctly folded proteins [21,22]. We envisioned that chaperone function might be greatly enhanced through co-localization with the translational machinery. Accordingly, we increased the spatial proximity of DnaJ and a target protein by anchoring DnaJ to the protein translation stop site via DnaJ fusion to a sequence-specific RNA binding domain (KH) originating from the human Nova-1 protein (Figure 1A). The localization of DnaJ immediately downstream the translation termination site will help the chaperone-target protein interaction occurred before the native co-translational folding completed. The binding of DnaJ-KH to its cognate 3′UTR hairpin loop was confirmed using a gel retardation assay (Supplementary Figure S1). We first applied the CRAS system to solubilize the aforementioned highly aggregation-prone recombinant proteins (ScFv, BR2-ScFv, UGD, UbiC, BMP2, Leptin, Adh1p, and HIV1-Pr) expressed in *E. coli* BL21(DE3) strain. The identities of expressed recombinant proteins was confirmed by Western Blot analysis using anti-His tag antibody, and their relative solubility is shown in Figure 1B and Supplementary Figure S2. The amino acid sequences of these POIs were scanned for DnaK binding sequence using Limbo [38] and predicted for the possible disulfide formation using DISULFIND [39]. Since DnaK and DnaJ share the majority of binding sites in *E. coli* [40], this analysis can be used to assess the potential binding of DnaJ to the POIs. Interestingly, while most of the POIs showed DnaK binding sites, HIV1-Pr does not contain any DnaK binding sequence (Supplementary Table S3). 

To evaluate the insoluble protein fraction, we examined protein expression by 10% SDS-PAGE. The POI expression was analyzed 4 h after induction with 0.5 mM Isopropyl-β-D-thiogalactopyranoside (IPTG). The ratio of insoluble and soluble protein fractions was calculated from SDS-PAGE images using ImageJ software [41] (Figure 1C). Even though Western Blot is a semi-quantitative method to estimate the level of protein expression, when examining the whole cell lysate that contains numerous types of proteins, we found that the results obtain by SDS-PAGE were more consistent. Therefore, all the image-based calculations in this work are generated from SDS-PAGE results. It is shown that ScFv, BR2-ScFv, and UGD solubility was markedly enhanced relative to the highly insoluble expression obtained when expressed individually or co-expressed without DnaJ-KH spatial constraint (Figure 1B,C). The significant difference of target recombinant protein solubility between mRNA non-coupling (simple target protein and chaperone co-expression) and mRNA coupling (chaperone co-localized to translation termination) systems partly supports our hypothesis that targeting DnaJ to the site of the translation could prevent the misfolding and aggregation from the early stage. Nevertheless, the CRAS system showed limited effects towards solubilizing the remaining four proteins, HIV1-Pr, UbiC, Leptin, and BMP2 (Figure 1C). It is noteworthy that even though the overexpression of DnaK might inhibit cell growth [42], we did not observe any retarded growth of *E. coli* cells in the experiments.

**Figure 2:**
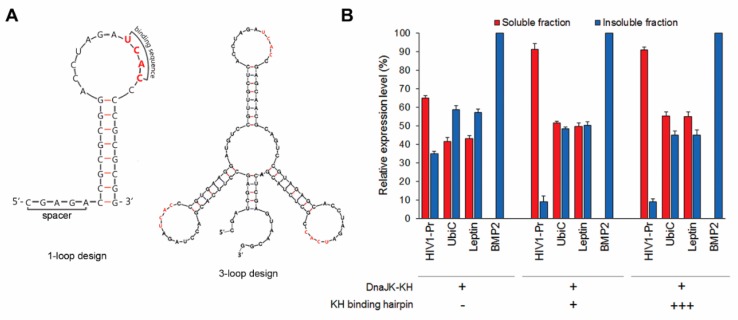
Combinatorial effect of increasing 3′UTR KH hairpin number and employing the chimeric chaperone fusion DnaJ-DnaK-KH (DnaJK-KH) on solubilizing four recombinant proteins, HIV1-Pr, UbiC, leptin, and Adh1p. (**A**) Sequence designs for constructing the 1-loop and 3-loop KH hairpin in the target protein mRNA 3′UTRs using RNA Designer and mFold. The spacer described in 1-loop design is a sequence between the stop codon of a gene encoding a target protein and the 3′UTR hairpin, varied from 5- to 30-nt. (**B**) The solubility of the four recombinant proteins that were not solubilized using the CRAS system with DnaJ-KH as the main chaperone, utilizing a chimeric DnaJK-KH chaperone including one (+) or three (+++) 3′UTR sequences downstream of the target gene coding sequence. Soluble protein quantification was conducted using ImageJ v1.48 software on Coomassie Blue-stained SDS-PAGE images. Error bars represent ± S.D. from three independent experiments.

As multiple ribosomes are concurrently involved in bacterial translation (i.e., polysomal translation), the CRAS system with one chaperone anchored to one mRNA may be insufficient for preventing certain target protein aggregation. Stoichiometric chaperone and substrate balance are critical for protein solubilization, especially during late growth phases. Therefore, to facilitate chaperone availability, multiple (up to 3) 3′UTR hairpin loops were introduced. The desired hairpin loops structure formation and a separation of the stop codon and first hairpin loop by 5- to 30-nt were engineered with computational simulations (Figure 2A). To our surprise, neither factor significantly affected target protein solubility up to 4 h after induction (Supplementary Figures S3 and S4). Contrary to our initial thought, the 30-nt distance between the 3′UTR loop and the translational stop site is still adequate for chaperone—substrate interaction, emphasizing the flexibility of the mRNA transcript as the scaffold. Furthermore, the long distance (up to 30-nt) between the translational stop site and the hairpin seem to not affect the expression level of the POI. Interestingly, the level of ScFv solubility expressed by the 3-loop design consistently retained high levels during protein expression, unlike the gradual reduction upon single 3′UTR hairpin loop use (Supplementary Figure S4). That reflects the unbalanced protein: chaperone ratio in the later growth phase with accumulated recombinant proteins albeit limited local chaperone number and turn-over rate. The higher number of DnaJ-KH molecules anchoring to the 3′UTR in 3-loop design may compensate for that phenomenon and maintain the high solubility of the target protein throughout the protein expression period.

### 2.2. DnaJ Can Function as the Sole Chaperone in CRAS System

To test the efficacy of DnaJ chaperone anchoring to the translation site, we deleted native *dnaJ* or *dnaK* genes from the *E. coli* genome and repeated CRAS system-mediated ScFv solubilization. As expected, no solubilization efficiency change was observed for Δ*dnaJ*, because most of the DnaJ molecules that effectively interact with ScFv were expressed from the plasmid pAMT7. However, the protein solubilization effect of CRAS system may be suppressed in the Δ*dnaK* strain lacking the major foldase chaperone. Conversely, this strain yielded slightly incremented ScFv solubility (Supplementary Figure S5). DnaJ functions primarily as a holdase, identifying and presenting misfolded proteins to foldase chaperones (e.g., DnaK and GroESL) but not directly refolding them [22]. However, DnaJ also exhibits foldase activity *in vitro* and facilitates proper folding in the absence of DnaK [43]. To determine whether DnaJ-KH in our system might solubilize protein without other chaperones, we simultaneously translated Adh1p and DnaJ-KH *in vitro* using the PURExpress kit (New England Biolab, Ipswich, MA, USA). DnaJ-KH solubilized Adh1p up to 50%, which was further improved to approximately 80% when the 3′UTR KH hairpin was introduced (Supplementary Figure S6). Unlike lysate-based *in vitro* translation kits, the PURExpress *in vitro* translation kit comprises only the translation components [44]. Therefore, the result suggests that the observed enhanced solubility of Adh1p arose solely from DnaJ activity. That can be explained by the binding of DnaJ to the nascent proteins prior to the translation termination could prevent unwanted misfolding and aggregation, reducing the formation of the inclusion body.

### 2.3. DnaJ-DnaK Chimeric Chaperone Enhances the Efficiency of The Native DnaK System

As the chaperone-mediated folding process involves multiple reactions, CRAS system activity may be further improved by co-localizing DnaJ with other chaperone proteins. That may facilitate the transfer of protein intermediates to downstream chaperones and DnaJ turnover. DnaK interacts with DnaJ to receive the misfolded protein or directly binds to the substrate for the refolding reaction, even though with narrower substrate range than that of DnaJ [40]. Therefore, we introduced DnaK sequence between the DnaJ and KH3 domains via two flexible linkers (GGGGS)_3_ and (GGGGS)_2_, respectively, to form a chimeric DnaJ-DnaK-KH chaperone (DnaJK-KH). The flexible linkers were used to prevent the steric hindrance between the fusion partners and ensure the fusion is fully functional. 

Accordingly, DnaJK-KH dramatically improved the solubility of HIV1-Pr, UbiC, and Leptin, yielding about 90% expressed HIV1-Pr and half of expressed UbiC and Leptin as soluble fractions (Figure 2B). The sudden increment of protein solubility is, interestingly, attributed mainly by the holding or folding activity of the DnaJK-KH fusion itself, as the majority of the improvement was shown without the KH binding hairpin (Figure 2B). The presence of the 3′UTR hairpin in single and triplet augmented the solubilization effect of DnaJK-KH, suggesting that the mRNA scaffold design can support even large chaperone complexes. Nonetheless, the differences were not as significantly as when a simple chaperone is exploited. The improvement in solubilization effect by utilizing DnaJK-KH and 3-loop design cannot improve the solubility of BMP2, though (Figure 2B).

### 2.4. In Vivo Monitoring of Chaperone Reactions

**Figure 3:**
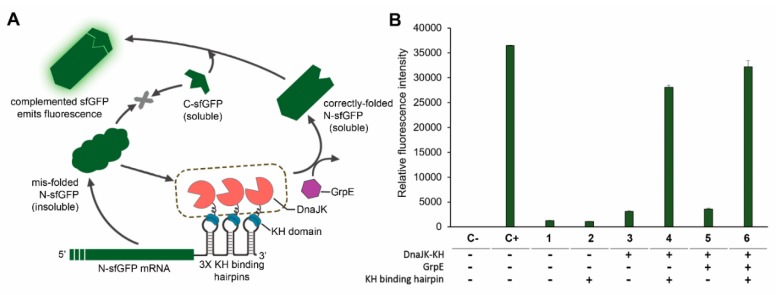
Monitoring CRAS system *in vivo* solubilization activity. (**A**) Scheme representing a split GFP experiment to monitor CRAS system *in vivo* protein solubilization activity: A superfolder GFP (sfGFP) is split in two fragments with a highly insoluble N terminal part (N-sfGFP) and soluble C terminal part (C-sfGFP). Introducing three KH binding hairpin loop repeats to the N-sfGFP mRNA 3′UTR targets this sfGFP part for solubilization by the DnaJK-KH CRAS system. Solubilized N-sfGFP complements C-sfGFP, emitting fluorescence. (**B**) *in vivo* N-terminal sfGFP solubilization using the CRAS system; fluorescence intensity of *E. coli* BL21(DE3) cells harboring the indicated CRAS system components including DnaJK-KH, KH binding hairpin, and GrpE, was determined at 488 nm λ_exc_ and 530 nm λ_em_. C− is negative control (empty plasmid) and C+ is positive control (expresses full-length sfGFP). The illustrated GrpE in (**A**) is either expressed from *E. coli* genome (GrpE was not presented in the expression plasmid), indicated as (−), or overexpressed from a plasmid, indicated as (+). Samples 1–6 show different combinations of the sfGFP expression and CRAS units, as indicated by the absence, shown by the symbol (−), and presence, shown by the symbol (+), of each component. Error bars in (**B**) represent ± s. d. from three independent experiments.

The high solubility of an expressed protein does not guarantee the high functionality as the protein may not be correctly folded or bound to other molecules, especially the unreleased chaperones. To monitor functional target protein folding *in vivo* and validate the efficacy of our method, we designed a green fluorescence complementary assay in which a superfolder green fluorescence protein (sfGFP) was divided into N- and C-terminal fragments, named as N-sfGFP and C-sfGFP respectively (Figure 3A). While the C-sfGFP is highly soluble, the N-sfGFP is aggregation-prone that 70–80% of the fragment expressed in the inclusion body (Supplementary Figure S7). Fluorescence level, hence, reflected CRAS-solubilized, functional N-sfGFP as a low solubility target protein. The non-coupling DnaJK-KH co-expression with N-sfGFP and C-sfGFP resulted in the doubled *in vivo* fluorescence level. However, it was only 8% the fluorescence level of the positive control, the full-length sfGFP. In contrast, concomitant 3-hairpin loop introduction to the N-sfGFP 3′UTR to recruit DnaJK-KH significantly increased fluorescence intensity to approximately 9-fold, equivalent to approximately 77% intensity of full-length sfGFP (Figure 3B). Compared to the fluorescence level of the split sfGFP system without CRAS system, the result is translated to a roughly 19-fold increase in the activity of N-sfGFP. Thus, the CRAS system increases functionally active protein expression, as well as solubility.

### 2.5. Co-Translational CLEX System Refolding Activity Solubilizes Aggregation-Prone Recombinant Proteins

**Figure 4:**
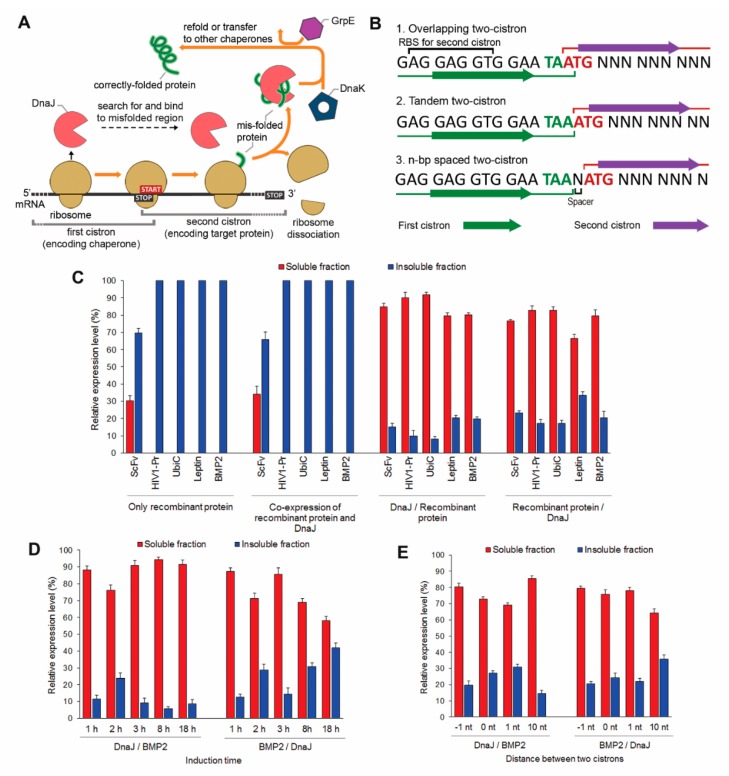
Recombinant protein solubilization by the CLEX system. (**A**) Schematic representation of CLEX system-mediated *in vivo* protein solubilization: the translationally coupled two-cistron expression system was utilized for translating DnaJ and a target protein in proximity, from a single mRNA transcript. A first cistron stop codon, TAA, is placed overlapping, in tandem, or n-nt preceding the second cistron start codon, TAG, illustrated in (**B**). In the first cistron, 6 nucleotides in the 3′ sequence serve as a ribosome-binding site (RBS) for the second cistron. Second cistron translation initiation involves both *de novo* translation and continuous translation from the first cistron. (**C**) CLEX system-mediated solubilization of five selected aggregation-prone recombinant proteins with chaperone DnaJ as the first or second cistron. Effect of DnaJ-target protein order (**D**) and distance (**E**) in the translationally coupled two-cistron expression construct were evaluated using BMP2. Slash symbols between proteins indicate the order of genes encoding each protein in the mRNA. The illustrated GrpE in (**A**) is either expressed from *E. coli* genome or overexpressed from a plasmid. Relative expression was assessed by SDS-PAGE and qualified using ImageJ v1.48 software. Error bars in (**C**–**E**) represent ± S.D. from three independent experiments.

Alternatively, to demonstrate the significance of chaperone and substrate spatial constraint without limiting chaperone number around the translational machinery, we constructed the CLEX system, a unique translationally coupled two-cistron expression system for solubilizing highly insoluble proteins (Figure 4A). The two-cistron system has been used to produce eukaryotic proteins or toxic peptides in *E. coli* [45,46] by placing the target protein gene downstream of a short sequence favoring translation initiation. Overlapping first and second cistron stop and start codons (5′-TAATG-3′) forces the ribosome complex to translate the second cistron without dissociating from the transcript [47]. In the CLEX system, *dnaJ* is overlapped with a target protein gene using either cistron sequence, with the first cistron containing a ribosome-binding site (5′-AGGAGGT-3′) for enhancing the expression of the second cistron. Specific intergenic sequences provide an overlapping (5′-TAATG -3′), tandem (5′-TAAATG-3′), or n nt-spaced two-cistron module (Figure 4B).

The overlapping CLEX system effectively solubilized highly insoluble proteins that were partly or not solubilized at all by the CRAS system including BMP2, HIV1-Pr, UbiC, and Leptin, yielding 60% to over 90% recombinant proteins in soluble forms (Figure 4C). Successful protein solubilization can be explained by the rapid DnaJ interactions during and after substrate translation, minimizing aggregation and facilitating refolding. Moreover, the non-dissociation of ribosome in transition between cistron translations may maintain at least the 1:1 (chaperone: target protein) ratio during translation, providing sufficient chaperones to recognize and bind misfolded proteins.

### 2.6. Two-Cistron Ordering and DnaJ Represent Primary Factors in Determining CLEX Protein Solubilization Efficiency

Expression levels and time of action differences indicated that DnaJ function in the CLEX system was dependent on cistron order (Figure 4D). Chaperone expression in the first cistron led to a higher soluble fraction, potentially owing to the availability of the already translated chaperone prior to second cistron translation. Placing DnaJ as the second cistron, in spite of the reduced chaperone expression level, still promoted the chaperone-substrate interaction to a lesser degree since both the two proteins are continuously expressed and accumulated in a limited space. Similar to CRAS systems with one and three 3′UTR KH hairpin loops, the CLEX system with first cistron DnaJ maintained high substrate protein (BMP2) solubility through an extensive expression period (Supplementary Figure S8). However, the reverse order resulted in gradually decreased protein solubility (Figure 4D and Supplementary Figure S8). It was probably caused by the higher BMP2 expression rate from the first cistron resulted in the unbalanced substrate: chaperone ratio in the later growth phase. Notably, in both schemes, increased intergenic sequence up to 10-nt somewhat reduced chaperone-mediated substrate protein solubilization (Figure 4E). Even though the ratio of soluble BMP2 seemed to be increased by using 10-nt intergenic sequence in the DnaJ/BMP2 arrangement, the total expression level of BMP2 dropped significantly as the start codon was away from the 3′ termini of the first cistron. The higher solubility was the direct consequence of the low protein expression level (Supplementary Figure S9). The lower efficiency of the chaperone-mediated solubilization effect may be explained by the higher percentage of *de novo* translation from the second cistron, in which the interaction of proteins translated from the two cistrons is not promoted. These data suggest the importance of placing the two genes in proximity to maximize protein interaction.

Native DnaK function in solubilizing recombinant proteins in the CLEX system was also examined using the *ΔdnaJ* and *ΔdnaK* strains (Supplementary Figure S10). Similar to the data with CRAS system, the elimination of the native DnaJ or DnaK did not show any change in the solubility of BMP2, regardless of whether it was expressed as the first or second cistron with DnaJ. Again, in our second system, DnaJ was demonstrated to be capable as the sole chaperone, solubilizing most of the expressed BMP2. Remarkably, this POI was completely insoluble in CRAS system, even with the additional support from DnaK.

### 2.7. GrpE-Mediated DnaK Release from The Substrate Is Critical for Obtaining Functional Proteins

**Figure 5:**
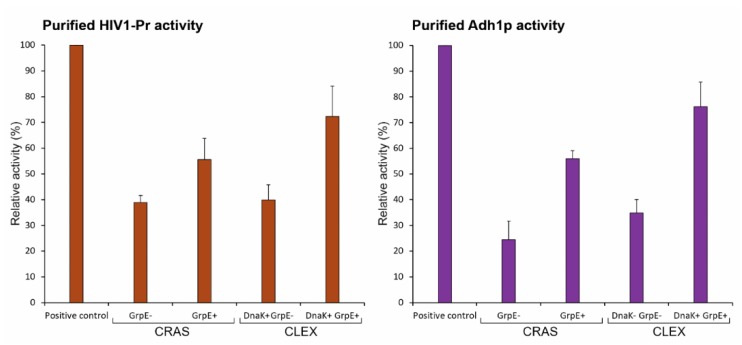
*In vitro* activities of purified (**A**) HIV-1 protease and (**B**) Adh1p expressed using CRAS and CLEX systems in the absence and presence of GrpE or GrpE and DnaK. His-tagged HIV-1 protease and Adh1p were purified using Ni-IDA resin from 1 l culture. Positive controls are the commercially available purified HIV-1 protease and Adh1p. Error bars show ± S.D. from three independent experiments.

For examining protein activity, we chose HIV-1 protease and Adh1p to purify and performed *in vitro* activity assays. Despite high protein solubility levels, HIV-1 protease and Adh1p purification using Ni-IDA resin resulted in relatively weak protein activity compared to that of commercial enzymes (Figure 5), indicating that the majority of soluble proteins were non-functional. SDS-PAGE analysis of purified protein bands indicated chimeric chaperone DnaJK-KH co-purification with target proteins (Supplementary Figure S11), suggesting tight chaperone-substrate binding. 

The adenine nucleotide exchange factor GrpE functions as a co-chaperone in the DnaJ chaperone system to promote substrate release from DnaK [48]. To achieve a better stoichiometry among DnaJ- DnaK-GrpE, improve the efficiency of the refolding process through substrate release, and potentially enhance protein functionality, we co-overexpressed GrpE along with other CRAS and CLEX system components. In the CLEX system, DnaK was also co-overexpressed in addition to GrpE as a high GrpE to DnaK ratio may cause cell division and growth defects [49]. In the CRAS system, GrpE addition was more effective than DnaJK-KH alone, as indicated by a further increased N-sfGFP and C-sfGFP complex fluorescence from 77% to 88% of the full-length sfGFP (Figure 3B). The addition of GrpE co-overexpression in CRAS and CLEX systems drastically enhanced HIV-1 protease and Adh1p enzyme activities by approximately 0.45- to 1.15-fold (Figure 5) and the DnaJK-KH was no longer found to be co-purified with the target protein (Supplementary Figure S11). The greater CLEX-mediated protein function enhancement was mediated by both DnaK and GrpE activities, whereas only GrpE was added to the CRAS system. It is notable that the co-overexpression of GrpE did not improve the solubility, but the activity of tested proteins. These results indicate the important role of GrpE in producing soluble and active proteins by either method, even though high protein purity is still required to approach commercial product activity levels and GrpE and DnaK requirements may vary depending on the target protein.

### 2.8. The CLEX System Is Not Efficient in Facilitating the Folding of Large Aggregation-Prone Proteins

**Figure 6:**
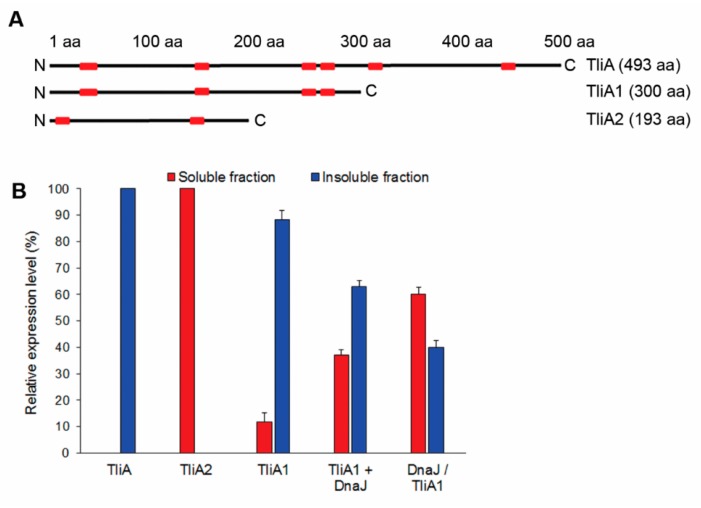
Size-dependent recombinant protein solubilization using the CLEX system. (**A**) Distribution of DnaJ and DnaK binding sequences on the lipase TliA is shown by the red rectangle positions. Binding sites were predicted using a Limbo chaperone binding site predictor [38]. (**B**) TliA1 fragment solubilization using the CLEX system. TliA1 + DnaJ indicates non-coupled co-overexpression, whereas DnaJ/TliA1 represents the CLEX system with DnaJ used as the first cistron. Relative expression was measured by SDS-PAGE and qualified using ImageJ v1.48 software. Error bars in (**B**) indicate ± S.D. from three independent experiments.

We achieved only marginally increased protein solubility when we applied our system to high-molecular-weight proteins, such as for the 52 kDa lipase TliA. As such large proteins tend to contain multiple misfolding-prone domains, our systems may not be sufficient to facilitate chaperone interaction with these multiple substrates prior to their irreversible misfolding. To test whether localizing the chaperone in proximity to internal misfolding regions of TliA is more effective, we, therefore, cleaved the *tliA* gene into two fragments, a highly insoluble (TliA1) and very soluble (TliA2) fragment, and applied our system to TliA1 (Figure 6A,B). 

The CLEX system with *dnaJ* expressed as the first cistron and *tliA1* as the second solubilized approximately 60% of TliA1 whereas non-coupled co-overexpression provided only 25% solubility (Figure 6B and Supplementary Figure S12). This result suggests that the insufficient exposure to chaperones of multiple internal misfolding regions in complex and large proteins limited the success of our systems. An advanced design to enforce more chaperones to interact with the polypeptide during the translation is critical to solubilize large and complex proteins.

## 3. Discussion

In this report, we introduced a novel approach to enhance the activity of DnaK molecular chaperone system on solubilizing recombinant proteins by spatially constraining the chaperones to the translation machinery. Either by anchoring the DnaJ or the chimeric DnaJK chaperones to the 3′UTR of the POIs’ mRNA (CRAS system) or coupling the translation of DnaJ and POIs (CLEX system), we surpassed the solubilization efficiency achieved by the overexpression of only DnaJ. Application of our two novel systems to solubilize different aggregation-prone recombinant proteins from various sources indicated that the high local concentration of molecular chaperones at the translation site is critical for fully exploiting protein folding activity. While CRAS system maintains a small number of chaperone molecules bound to the 3′UTR of POIs’ mRNAs, CLEX system continuously supplies chaperone molecules during the translation of these transcripts. Both systems were superior to the use of non-coupling DnaJ co-overexpression as in our results they showed significant increases in the solubility of POIs. 

It was reported that DnaJ and DnaK function individually as holdases to prevent the folding process, and while DnaJ binds to unfolded protein, DnaK recognizes partly folded states [50]. That explains the advantage of our system to use DnaJ as the first chaperone to interact early with nascent proteins, instead of using only DnaK [51,52]. Noticeably, with the support of our systems, the co-chaperone DnaJ can perform as the sole chaperone with remarkably high solubilization effect, regardless of the relatively weak functionality observed when it is not localized to the translation machinery. As when DnaJ and DnaK work in synergy, they form an effective foldase [50], the fusion of DnaJ and DnaK should exhibit higher efficiency in producing soluble proteins. Among the selected aggregation-prone proteins, 7 out of 8 were produced from 50-90% in the soluble fraction with CRAS employing DnaJK-KH chimeric chaperone, and 5 out of 5 were expressed from 70-90% in the soluble fraction when CLEX system with DnaJ was applied. The highly soluble expression of recombinant proteins in this work was achieved without requiring additional sequences such as solubility tags or fusion partners, although affinity tags for purification may be needed. These systems, hence, substantially reduce associated time and costs of downstream resolubilization, *in vitro* refolding, and protease-mediated cleavage. Moreover, chaperone-coupling systems, which produce proteins in their intact forms, benefit native intracellular environment protein functional studies and metabolic engineering.

The CLEX system outperforms the CRAS system regarding solubilization activity, suggesting that forcing chaperone-polypeptide interaction during translation is more efficacious. Nevertheless, the latter may be sufficient to prevent misfolding and aggregation for small proteins with few misfolding-prone domains. For simultaneous multi-protein solubilization, the CLEX system may achieve limited success as each protein requires cloning a *dnaJ* copy into a two-cistron system. Therefore, CLEX system is more suitable for producing single recombinant proteins, when the protein yield and simplicity of the system are of the highest priority. Conversely, the 3-loop CRAS system with DnaJK-KH may enhance multiple co-expressed protein solubility by simply introducing the 3′UTR KH-binding hairpin sequence to each mRNA. Thus, CRAS system is well suited for increasing the fraction of soluble, functional small proteins or optimizing metabolic pathways with more active enzymes. The design of CRAS system in this work is, unfortunately, not an ideal one, as the RNA binding affinity of KH3 domain, with K_D_ ≈ 500 nM, is much weaker than the 1–2 nM dissociation constants of some other RNA binding proteins like MS2 or PP7 [23,53,54]. Higher solubilization efficiency is, hence, expected with the optimized pairs of RNA binding domains and their cognate RNA structure. The use of Nova-1 KH3 in this work, albeit the weak RNA binding affinity, is sufficient to demonstrate the concept of an mRNA scaffold to promote the efficiency of a post-translational process like the chaperoning activity. Coupling the chaperone activity and the translation process, in fact, already exists in nature, as the Trigger factor (TF) is known to directly bind to the ribosome and shield the nascent polypeptides from protease and aggregation [55]. The chaperones used in our systems play an enforcement role to TF function, emphasizing the importance of holdases to prevent the misfolding and aggregation from early folding stages. An alternative design of our system with TF replacing DnaJ is also promising as well. Moreover, the increased activity of Adh1p and HIV1-Pr expressed from our systems with the addition of GrpE suggests that the use of the complete DnaK system is necessary for ensuring the functionality of the soluble proteins. The DnaK chaperone system was chosen for our CRAS and CLEX designs for its wide substrate range, rather simple tertiary structures, and as the best-characterized system to date. However, DnaK system disadvantages include decreased activity toward large proteins [56], potentially limiting large protein refolding efficiency of our systems, and proper stoichiometry requirement of all three components, DnaJ, DnaK, and GrpE [57,58]. Therefore, although our systems were effective with rather simple designs, further combinations with other chaperones, heat shock proteins, and balanced control of co-chaperone expression will likely expand the substrate range.

Last but not least, within the scope of this study, we are not tackling the issues of post-translational modifications of the proteins, especially the disulfide bond formation. Despite the popularity of *E. coli* as the host for producing recombinant proteins [59], the reducing cytoplasm of this enterobacteria does not support the formation of disulfide bonds [60]. Some POIs in this study require disulfide bonds to achieve correct tertiary structures (Supplementary Table S3), hence limiting the efficiency of our methods. To produce disulfide bond-containing recombinant proteins *in vivo* in their fully functional form, combining our strategies with other methods is necessary. These methods are readily available and described elsewhere, such as targeting a POI to the periplasm [61], co-expressing a POI with sulfhydryl oxidase [62], fusing a POI with thioredoxin [63], or using alternative *E. coli* hosts [64,65]. 

## 4. Materials and Methods

### 4.1. Bacterial Strains, Enzymes, and Chemicals

We utilized *Escherichia coli* strains XL1-Blue (Stratagene, La Jolla, CA, USA) and BL21(DE3) (Novagen, Madison, WI, USA) for all cloning experiments and gene expression, respectively. P1 transduction was used to construct strains lacking *dnaJ* and *dnaK* genes as previously described [66]. BW25113 strains with single knockouts (Δ*dnaJ* and Δ*dnaK*), obtained from the Keio collection [67], served as BL21(DE3) donor strains. The deletion strains were screened via colony polymerase chain reaction (PCR) using a primer pair flanking the target DNA region (Supplementary Table S1). Subsequently, the kanamycin resistance cassette was removed from the integrated host genome region using FLP recombinase expressed from pCP22 [68]. All utilized oligonucleotides (Genotech, Daejeon, South Korea) are listed in Supplementary Table S1. All chemicals were from Sigma-Aldrich (Steinheim, Germany).

### 4.2. Construction of Expression Vectors Encoding Chaperones and Target Recombinant Proteins

To construct a medium-copy number vector based on pACYCDuet-1 with two strong T7 promoters (Novagen), we replaced a low- (p15A) with a medium- (pBR322) copy number origin of replication, generating pAMT7. Chaperone genes (*dnaJ*, *dnaK*, and *grpE*) were PCR amplified from BL21(DE3) genomic DNA. *dnaJK* fusion was constructed by fusing *dnaK* downstream the *dnaJ* with a flexible (GGGGS)_3_ linker in between, using recombinant PCR. For high-level *dnaJ*, *dnaJ-KH*, and *dnaJK-KH* expression, a synthetic ribosome-binding site (RBS) was designed using the RBS calculator [69] and incorporated into the DnaJNcoF primer 5′-terminus. *grpE* was cloned downstream *dnaJK-KH*, under the control of a separated T7 promoter. The Nova-1 KH3 RNA-binding domain (KH) gene [70] with *E. coli* expression-optimized sequence was synthesized (Bioneer, Daejeon, South Korea) and fused downstream of either the *dnaJ* or *dnaJK* fusion by recombinant PCR. A flexible (GGGGS)_2_ linker was used in between KH3 and DnaJ or DnaK. Target recombinant protein genes were PCR amplified from various sources: *scfv* and *br2-scfv* from pBR2ScFv [25]; *ugd* and *ubiC* from BL21(DE3) genomic DNA; and *adh1p* from *Saccharomyces cerevisiae* genomic DNA. The *hiv1-pr* gene [71] was codon-optimized and constructed by recombinant PCR; and *tliA* was amplified from pTliA [72]. For the GFP complementation assay, a superfolder green fluorescent protein, sfGFP (synthesized by Bioneer) served as a template for N- and C-termini sfGFP amplification. A synthetic RBS for high-level sfGFP and N-terminal sfGFP expression was designed and incorporated into the sfGFPXbaF primer 5′-terminus. Primers are listed in Supplementary Table S1. For purification and Western Blot analysis, a 6 × His-tag was added to the N-terminus of the forward primer (for HIV1-Pr) or to the C-terminus of the reverse primer (for the remaining target recombinant proteins). A synthetic RBS for high-level HIV1-Pr expression was designed and incorporated into the HIVPrXbaF primer 5′-terminus. PCR products and their corresponding expression vectors were digested using restriction enzymes (Supplementary Table S1), then ligated to pAMT7 (chaperones) or pET16b (recombinant proteins).

### 4.3. Construction of RNA Scaffolds

The RNA scaffold system KH domain binding loop sequence was designed using RNA Designer and mFold [73,74] with sequence constraints of 5′-NNNNNNNNACCTAGATCACCNNNNNNNN-3′, where N represents any nucleotide for the 8-bp stem structure and the underlined sequence represents the KH domain binding loop sequence. A 0-, 5-, or 30-nt spacer is used to separate the loop sequence and the translation stop codon. For the multiple binding loop RNA scaffold system, a 5-nt random spacer was added between individual stem-loop structures. Stem-loop structure assembly to target protein genes was conducted by PCR using reverse primers containing the stem-loop structure sequence in the 3′UTR (Supplementary Table S1).

### 4.4. Construction of the CLEX System

The DnaJ chaperone and recombinant protein (ScFv, UbiC, Leptin, HIV1-Pr, BMP2, and TliA) in the CLEX system were constructed in two different arrangements, with DnaJ as the first or second cistron. A 12-nt (5′-GAGGAGGTGGAA-3′) region encoding amino acid sequence (EEVE) including a Shine-Dalgarno (SD) sequence (underlined), was introduced into the C-terminus of the first cistron to improve second cistron gene translational initiation [75]. Furthermore, to ensure translational coupling and promote DnaJ and recombinant protein interaction, the first cistron termination codon overlapped with the second cistron initiation codon by 1-nt (5′-TAATG-3′) [75]. DnaJ chaperone and recombinant protein assembly for CLEX system construction was conducted by recombinant PCR (primer sequences in Supplementary Table S2). PCR products and pET16b were restriction digested (Supplementary Table S2) and ligated to the expression vector. Note that even though pET16b and pAMT7 share the same origin of replication, pBR322, their antibiotic resistances are different (ampicillin and chloramphenicol, respectively). Thus, the two plasmids can co-exist stably within the experiment period, as long as both the antibiotic pressures are maintained [76,77].

### 4.5. Protein Solubilization Test

*E. coli* BL21(DE3) co-transformed with chaperone and target recombinant protein plasmids were inoculated into 3 mL lysogenic broth (LB) medium (10 g L^−1^ tryptone, 5 g L^−1^ yeast extract, and 10 g L^−1^ sodium chloride) supplemented with ampicillin and chloramphenicol (50 and 25 µg mL^−1^ final concentration, respectively) and incubated in a rotary shaker (200 rpm) at 37 °C overnight. Then, 1 mL overnight culture was inoculated into 100 mL LB medium supplemented with ampicillin and chloramphenicol (50 and 25 µg mL^−1^ final concentration, respectively) and grown at 37 °C (200 rpm). At OD_600_ = 0.5–0.6, chaperone and target protein expression were induced with 0.5 mM IPTG for additional 4 h incubation. Subsequently, cells (3 mL) were centrifuged for 1 min at 4 °C and 16,000 × g, resuspended in 10 mM Tris-EDTA (TE) buffer at pH 7.6 (500 µL) and lysed by sonication. Soluble and insoluble fractions were separated by centrifugation at 21,600× *g* for 15 min at 4 °C. The insoluble pellet was washed twice in 1% Triton X-100 and resuspended in 10 mM TE buffer (pH 7.6) (insoluble fraction). Target recombinant protein solubility was examined using sodium dodecyl sulfate polyacrylamide gel electrophoresis (SDS-PAGE). Relative solubility of a target recombinant protein was calculated by comparing whole cell lysate and target recombinant protein soluble fraction band intensities from Coomassie Blue stained SDS-PAGE gels using ImageJ software [41].

### 4.6. Western Blot Analysis

After the electrophoresis, proteins separated on the gel were transferred onto a nitrocellulose membrane (pore size 0.2 µm, Bio-Rad, Hercules, CA, USA) for 120 min using the Trans-blot SD semi-dry electrophoretic transfer cell (Bio-Rad) at 70 V (voltage constant). The membrane was blocked for 1 h at room temperature with 5% skim milk (Wako Pure Chemical Industries) in Phosphate buffered saline containing 0.1% Tween-20 (PBS-T). The membrane was then incubated for 8 h at 4 °C with a primary antibody, mouse anti-His tag antibody (Cell Signaling Technologies, Danvers, MA, USA), at 1:1000 dilution in PBS-T containing 5% skim milk. After washing three times with PBS-T for 15 min, the membrane was incubated for 1 h at room temperature with a secondary antibody, HRP-conjugated anti-mouse IgG antibody (Cell Signaling Technologies), diluted by 1:1000 ratio in PBS-T containing 5% skim milk. The membrane was washed three times with PBS-T for 15 min and then treated with Western Blot detection solution A and B of Pico EPD Kit (ELPIS Biotech, Daejeon, South Korea) by 1:1 ratio for visualizing protein bands. Photographs of blots were taken by the Chemidoc XRS+ illumination system (Bio-Rad).

### 4.7. Protein Solubility Test with in vitro Translated Proteins

pET16b-Adh and pAMT7; pET16b-Adh3L and pAMT7; pET16b-Adh and pAMT7-DnaJ-KH; pET16b-Adh3L and pAMT7-DnaJ-KH were used as *in vitro* translation templates with the PURExpress^®^ *In Vitro* Protein Synthesis Kit. After 4 h incubation at 37 °C, 25 µL reaction mix was diluted using 75 μL TE buffer (10 mM) (pH 7.6). Soluble and insoluble fractions were separated by centrifugation and target recombinant protein solubility was examined by SDS-PAGE as for *in vivo* expressed proteins.

### 4.8. Purification of DnaJ-KH Fusion Proteins

*E. coli* BL21(DE3) harboring pAMT7-DnaJ-KH was cultured in LB medium supplemented with chloramphenicol, induced for protein expression, 20 mL cells harvested, resuspended in 1 × native IMAC lysis buffer (Bio-Rad), and lysed by sonication. Cleared cell lysate was centrifuged at 21,600× *g* for 15 min at 4 °C. DnaJ-KH was purified from the soluble fraction using the automated ProfiniaTM protein purification system (Bio-Rad) Native IMAC method according to manufacturer recommendation. Purified DnaJ-KH was then dialyzed against phosphate buffered saline (PBS) pH 7.4 and 25 mM Tris-HCl buffer (pH 8.8) containing 5 mM dithiothreitol, 100 mM NaCl, and 10% glycerol.

### 4.9. GFP Complementation Assay

Correctly folded recombinant protein formation following CRAS system application was evaluated based on the previously reported GFP complementation assay [78] with some modifications. *E. coli* BL21(DE3) harboring pAMT7-DnaJK-KH and pET16b-sfGFP/pET16b-CsfGFP-NsfGFP/pET16b-CsfGFP-NsfGFP3L was cultured in LB medium supplemented with ampicillin and chloramphenicol, induced for protein expression, 1 mL cells was harvested, resuspended in 500 µL PBS (pH 7.4), diluted to OD600 of 1, and loaded into a 96-well black plate (SPL Life Sciences, Gyeonggi-do, South Korea). Fluorescence intensity was determined for each well (λ_exc_ = 488 nm/λ_em_ = 530 nm) using the Infinite F200 PRO instrument (Tecan, Männerdorf, Switzerland).

### 4.10. Electric Mobility Shift Assay

Anti-p21ras-ScFv mRNAs without or with 1 and 3 binding loops were generated using a HiScribe™ T7 High Yield RNA Synthesis Kit (New England Biolabs). Then, 200 µM purified DnaJ-KH was mixed with 0.1 nM mRNA in PBS pH 7.4, incubated at 25 °C for 30 min, and analyzed by 2% agarose gel electrophoresis in Tris-boric acid-EDTA buffer for 20 min at a constant 50 V. The gels were visualized using a Gel-Doc gel documentation system (Bio-Rad).

### 4.11. Activity Assay of Alcohol dehydrogenase 1 (Adh1p)

*E. coli* BL21(DE3) harboring pET16b-Adh3L and pAMT7-DnaJK-KH-GrpE or pET16b-Adh3L and pAMT7-DnaJK-KH-GrpE (CRAS system); or pET16b-AdhDnaJ and pAMT7 or pET16b-AdhDnaJ and pAMT7-DnaK-GrpE (CLEX system) was cultured in 3 mL LB medium and induced for protein expression, and 100 mL cells harvested as described above. Cells were resuspended in 1 × binding buffer (0.5 M NaCl, 5 mM imidazole, 20 mM Tris-HCl, pH 7.9), and lysed by sonication. Cleared cell lysate was then centrifuged at 21,600× *g* for 15 min at 4 °C. Proteins in the supernatants were purified using Hi-Bind Agarose Resin (Ni-IDA) (ELPIS Biotech, Daejeon, Korea) according to manufacturer recommendation. Purified Adh1p was then dialyzed against 20 mM Tris-HCl buffer (pH 8.0) containing 1 mM dithiothreitol, 200 mM NaCl, and 10% glycerol. Purified Adh1p activity (1 µg) from the eluted fraction was compared to that of 1 µg recombinant alcohol dehydrogenase (Sigma-Aldrich) using the Alcohol Dehydrogenase Activity Assay kit (Sigma-Aldrich) according to manufacturer recommendation.

### 4.12. Activity Assay of HIV-1 Protease (HIV1-Pr)

*E. coli* BL21(DE3) harboring pET16b-HIVpr and pAMT7-DnaJK-KH-GrpE or pET16b-HIVpr3L and pAMT7-DnaJK-KH-GrpE (CRAS system); or pET16b-HIV1PrDnaJ and pAMT7 or pET16b-HIV1PrDnaJ and pAMT7-DnaK-GrpE (CLEX system) was cultured and induced for protein expression in LB medium, then purified using Ni-IDA resin as described for preparing Adh1p samples. Purified HIV1-Pr activity (0.2 µg) from the eluted fraction was compared to that of 0.2 µg recombinant HIV1-Pr (AnaSpec, Fermont, CA, USA) using The SensoLyte^®^ 520 HIV Protease Assay Kit (AnaSpec, Fermont, CA, USA) according to manufacturer recommendation.

## 5. Conclusions

Overall, both mRNA engineering approaches presented here support the concept of coupling translation and folding activity with spatial constraints to promote functionally active soluble protein production. These strategies represent economical methods to ensure higher chances of solubilizing aggregation-prone recombinant proteins, providing more power to available chaperone systems and reducing their reliance on inefficient posttranslational procedures. The current systems are limited to the water-soluble expression of medium-length proteins (less than 44 kDa); however, various factors can be adjusted and properly controlled to apply the principle of these methods to larger aggregation-prone proteins. 

## Supplementary Materials

Supplementary materials can be found at https://www.mdpi.com/1422-0067/20/13/3163/s1.
