# Supplementary material for: mRNA Engineering for the Efficient Chaperone-Mediated Co-Translational Folding of Recombinant Proteins in Escherichia coli
Source: Int J Mol Sci. 2019 Jun 28;20(13):3163. doi: 10.3390/ijms20133163 (PMC6651523; doi:10.3390/ijms20133163)
Supplement: Supplementary file 1 [file ijms-20-03163-s001.pdf]

*Supplementary information*

# mRNA engineering for the efficient chaperone-mediated co-translational folding of recombinant proteins in *Escherichia coli*

**Le Minh Bui [1,2,†], Almando Geraldi [3,†], Thi Thuy Nguyen [3], Jun Hyoung Lee [1], Ju Young Lee [4], Byung-Kwan Cho [1,3,5,*] and Sun Chang Kim [1,3,5,*]**

[1] KAIST Institute for BioCentury, Korea Advanced Institute of Science and Technology (KAIST), Daejeon 34141, South Korea; junhlee@kaist.ac.kr (J.H.L.)

[2] NTT Hi-Tech Institute, Nguyen Tat Thanh University (NTTU), Ho Chi Minh City 700000, Vietnam; blminh@ntt.edu.vn (L.M.B.)

[3] Department of Biological Sciences, Korea Advanced Institute of Science and Technology (KAIST), Daejeon 34141, South Korea; almandogeraldi@kaist.ac.kr (A.G.); nguyenthuy@kaist.ac.kr (T.T.N.)

[4] Center for Bio-based Chemistry, Korea Research Institute of Chemical Technology (KRICT), Ulsan, 44429, South Korea; juylee@krict.re.kr (J.Y.L.)

[5] Intelligent Synthetic Biology Center, Korea Advanced Institute of Science and Technology (KAIST), Daejeon 34141, South Korea

* Correspondence: sunkim@kaist.ac.kr (S.C.K.), bcho@kaist.ac.kr (B.-K.C.); Tel.: +82-42-350-2619 (S.C.K.); Tel: +82-42-350-2620 (B.-K.C.)

† These co-first authors contributed equally to this work.

**Supplementary Table S1. Primers used for constructing CRAS system**

| Primer name | Sequence 5′ → 3′ |
|---|---|
| DnaJNcoF | TGATAACCATGGAAGATTCTACGGTTAACACAATGGCTAAGCAAGATTATTACG |
| DnaJXhoR | ATTTCACTCGAGGCGGGTCAGGTCGTCAAA |
| KHNcoF1 | TAATGACCATGGAAACCGACGGTTCTAAAGACGTTGTTGAAATCGCTGTTCCGGAAAA CCTGGTTGGTGCTATCCTGGGTAAAGGTGGTAAAAC |
| KHRecR1 | GCCCGGAACAAATTCACCTTTTTTAGAAATCTGGATACGAGCACCTGTCAGTTCCTGGT ATTCAACCAGGGTTTTACCACCTTTACCCAGGA |
| KHRecF2 | AAAAGGTGAATTTGTTCCGGGCACCCGTAACCGTAAAGTTACCATCACAGGCACCCCG GCTGCTACCCAGGCTGCTCAGTACCTGATCACAC |
| KHXhoR2 | TTATCACTCGAGTTAACCAACTTTCTGCGGGTTAGCAGCACGAACACCCTGTTCGTAGG TGATACGCTGTGTGATCAGGTACTGAGCAGC |
| DnaJLinkR | GCTGCCGCCACCACCGCTACCGCCACCGCCGCGGGTCAGGTCGTCAAA |
| LinkKHF | GGTAGCGGTGGTGGCGGCAGCACCGACGGTTCTAAAGACGTT |
| KHXhoR | ATTTCACTCGAGTTAACCAACTTTCTGCGGGTT |
| DnaJXhoR | ATTTCACTCGAGGCGGGTCAGGTCGTCAAA |
| KH-6xHis-EcoR | CGATTAGGATCCTCATCATTAATGATGGTGGTGATGGTGAGATCCACGCGGAACCAGA CCAACTTTCTGCGGGTTAG |
| DnaJR | AGAACCTCCGCCGCCAGAACCCCCGCCACCGCGGGTCAGGTCGTCAAAAAA |
| DnaKF | TCTGGCGGCGGAGGTTCTGGTAAAATAATTGGTATCGACCTGG |
| DnaKR | GCTACCGCCACCGCCTTTTTTGTCTTTGACTTCTTCAAATTC |
| KHF | GACAAAAAAGGCGGTGGCGGTAGC |
| KHBamHR | CGATTAGGATCCTCATTATTAACCAACTTTCTGCGGGT |
| GrpENdeF | AGCTGACATATGAGTAGTAAAGAACAGAAAACGCC |
| GrpEXhoR | CGATTACTCGAGTCATTATTAAGCTTTTGCTTTCGCTACAG |
| ScFvNdeF | TGATAACATATGCAGGTCCAACTGCAGC |
| ScFvXhoR | TCATTACTCGAGTCATCATTAGTGGTGGTGGTGGTGGTGTTTGATCTCCAGCTTGGTCC |
| ScFv1LXhoR | CCGTTACTCGAGCCGCGCGGGGTGATCTAGGTCCGCGCGGTCGTCGTCGTCATCATTAG TGGTGGTGGTGGTGGTGTTTGATCTCCAGCTTGGTCC |
| 3L-1R | AGGTGAGCAACGGACATCCTTCACGGGTGATCTAGGTCGTGAAGGCTCGATCGTCATC ATTAGTGGTGGTGGTGGTGGTG |
| 3LXhoR | CCGTTACTCGAGTCGTAGAGCGGTGATCTAGGTGCTCTACGGACTGCGTTGCTCGGTGA TCTAGGTGAGCAACGGACATCCTTCACG |
| BR2ScFvNdeF | TGATAACATATGCGTGCTGGTCTGCAGT |
| UGDNdeF | TGATAACATATGAAAATCACCATTTCCGG |
| UGDXhoR | CCGTTACTCGAGTTATTAGTGGTGGTGGTGGTGGTGGTCGCTGCCAAAGAGATCG |
| UGD1LXhoR | CCGTTACTCGAGCCGCGCGGGGTGATCTAGGTCCGCGCGGTCGTCGTCGTCATCATTAG TGGTGGTGGTGGTGGTGGTCGCTGCCAAAGAGATCG |
| AdhNdeF | TGATAACATATGTCTATCCCAGAAACTCAAAA |
| AdhXhoR | CCGTTACTCGAGTTATTAGTGGTGGTGGTGGTGGTGTTTAGAAGTGTCAACAACGTATC T |
| Adh1LXhoR | CCGTTACTCGAGCCGCGCGGGGTGATCTAGGTCCGCGCGGTCGTCGTCGTCATCATTAG TGGTGGTGGTGGTGGTGTTTAGAAGTGTCAACAACGTATCT |
| UbiCNdeF | TGATAACATATGCGATTGTTGCGTTTTTGTTGC |
| UbiCXhoR | CCGTTACTCGAGTTATTAGTGGTGGTGGTGGTGGTACAACGGTGACGCCGGTA |
| UbiC1LXhoR | CCGTTACTCGAGCCGCGCGGGGTGATCTAGGTCCGCGCGGTCGTCGTCGTCATCATTAG TGGTGGTGGTGGTGGTGGTACAACGGTGACGCCGGTA |
| HIVPrXbaF | ATTCTAAATCTAGATTATTCACTACGCGTTAAGGAGGTACGACATGCACCATCACCACC ATCATCCTCAAATCACCCTGTGGC |

| | |
|---|---|
| HIVPrXhoR | CCGAATTACTCGAGTCATCATTAGAAGTTCAGGGTGCAACCGATCTGGGTCAGCATGTT ACGACCGATGATGTTGATCGGGGTCG |
| HIVPr1LXhoR | CCGTTACTCGAGCCGCGCGGGGTGATCTAGGTCCGCGCGGTCGTCGTCGTCATCATTAG AAGTTCAGGGTGCAACCG |
| HIVPr3L-1R | AGGTGAGCAACGGACATCCTTCACGGGTGATCTAGGTCGTGAAGGCTCGATCGTCATC ATTAGAAGTTCAGGGTGCAACCG |
| LepNdeF | TGATAACATATGGTGCCCATCCAAAAAGTCC |
| LepXhoR | CCGTTACTCGAGTTATTAGTGGTGGTGGTGGTGGTGGCACCCAGGGCTGAGGTC |
| Lep1LXhoR | CCGTTACTCGAGCCGCGCGGGGTGATCTAGGTCCGCGCGGTCGTCGTCGTCATCATTAG TGGTGGTGGTGGTGGTGGCACCCAGGGCTGAGGTC |
| BMP2NdeF | TGATAACATATGCAAGCCAAACACAAACAG |
| BMP2XhoR | CCGTTACTCGAGTTATTAGTGGTGGTGGTGGTGGTGGCGACACCCACAACCCTC |
| BMP21LXhoR | CCGTTACTCGAGCCGCGCGGGGTGATCTAGGTCCGCGCGGTCGTCGTCGTCATCATTAG TGGTGGTGGTGGTGGTGCGACACCCACAACCCTC |
| sfGFPNdeF | TGATAACATATGCAAGCCAAACACAAACAG |
| sfGFPEcoR | AGGTCAGAATTCTCATCATTACGTAATACCTGCCGCATTC |
| CsfGFPXbaF | CCATGATCTAGAAATAATTTTGTTTAACTTTAAGAAGGAGATATACCATGGTGCCCATC CAAAAAGTC |
| CsfGFPNdeR | CCGTTACATATGTCATCATTATGTAATCCCAGCAGCATTTAC |
| NsfGFPEcoR | AGGTCAGAATTCTCATCATTATTTTTCGTTCGGATCTTTAGACA |
| NsfGFP3L-1R | AGGTGAGCAACGGACATCCTTCACGGGTGATCTAGGTCGTGAAGGCTCGATCGTCATC ATTATTTTTCGTTCGGATCTTTAGACA |
| sfNGFP3LEcoR | AGGTCAGAATTCTCGTAGAGCGGTGATCTAGGTGCTCTACGGACTGCGTTGCTCGGTGA TCTAGGTGAGCAACGGACATCCTTCAC |

**Supplementary Table S2. Primers used for constructing CLEX system**

| Primer name | Sequence 5′ → 3′ |
|---|---|
| DnaJNdeF | TGATAACATATGGCTAAGCAAGATTATTACG |
| ScFvFpol | GTTTTTTGACGACCTGACCCGCGAGGAGGTGGAATAATGCAGGTCCAACTGCAGC |
| ScFvXhoR | TCATTACTCGAGTCATCATTAGTGGTGGTGGTGGTGGTGTTTGATCTCCAGCTTGGTCC |
| ScFvNdeF | TGATAACATATGCAGGTCCAACTGCAGC |
| DnaJFpol | CACCACCACCACCACCACGAGGAGGTGGAATAATGGCTAAGCAAGATTATTACG |
| DnaJXhoR | ATTTCACTCGAGTTATTAGCGGGTCAGGTCGTCAAA |
| UbiCFpol | GTTTTTTGACGACCTGACCCGCGAGGAGGTGGAATAATGCGATTGTTGCGTTTTTGTTGC |
| UbiCXhoR | CCGTTACTCGAGTTATTAGTGGTGGTGGTGGTGGTGGTACAACGGTGACGCCGGTA |
| UbiCNdeF | TGATAACATATGCGATTGTTGCGTTTTTGTTGC |
| HIVPrFpol | GTTTTTTGACGACCTGACCCGCGAGGAGGTGGAATAATGCACCATCACCACCATCATC CTCAAATCACCCTGTGGC |
| HIVPrXhoR | CCGAATTACTCGAGTCATCATTAGAAGTTCAGGGTGCAACCGATCTGGGTCAGCATGTT ACGACCGATGATGTTGATCGGGGTCG |
| HIVPrXbaF | ATTCTAAATCTAGATTATTCACTACGCGTTAAGGAGGTACGACATGCACCATCACCACC ATCATCCTCAAATCACCCTGTGGC |
| DnaJFpolHIVPr | CGGTTGCACCCTGAACTTCGAGGAGGTGGAATAATGGCTAAGCAAGATTATTACG |
| LepFpol | GTTTTTTGACGACCTGACCCGCGAGGAGGTGGAATAATGGTGCCCATCCAAAAAGTCC |
| LepXhoR | CCGTTACTCGAGTTATTAGTGGTGGTGGTGGTGGTGGTACAACGGTGACGCCGGTA |
| LepNdeF | TGATAACATATGGTGCCCATCCAAAAAGTCC |
| BMP2FPol | GTTTTTTGACGACCTGACCCGCGAGGAGGTGGAATAATGCAAGCCAAACACAAACAG |
| BMP2XhoR | CCGTTACTCGAGTTATTAGTGGTGGTGGTGGTGGTGGCGACACCCACAACCCTC |

| | |
|---|---|
| BMP2NdeF | TGATAACATATGCAAGCCAAACACAAACAG |
| TliANdeF | TGATAACATATGCATCATCATCATCATCATCATCATCACAGCA |
| TliAEcoR | CTGAGAATTCTCATCATTAACTGATCAGCACACCCTCGCTCC |
| TliAFPol | GTTTTTTGACGACCTGACCCGCGAGGAGGTGGAATAATGCATCATCATCATCATCATCA TCATCACAGCA |
| TliAEcoR | CTGAGAATTCTCATCATTAACTGATCAGCACACCCTCGCTCC |
| DnaJTliAFpol | GGAGCGAGGGTGTGCTGATCAGTGAGGAGGTGGAATAATGGCTAAGCAAGATTATTAC G |
| DnaJEcoR | CTGAGAATTCTTATTAGCGGGTCAGGTCGTCAAA |
| TliA1EcoR | CCGTTAGAATTCTCATCATTAGTCGGTGGTCGACTCGTG |
| TliA1FPol | AGTTTTTTGACGACCTGACCCGCACCGGAGGTACATAATGCATCATCATCATCATCATC ATCATCACAGCA |
| TliA1EcoR | CCGTTAGAATTCTCATCATTAGTCGGTGGTCGACTCGTG |
| TliA2NdeF | TGATAACATATGAACATCGTCAGCTTCAACG |
| AdhNdeF | TGATAACATATGTCTATCCCAGAAACTCAAAA |
| DnaKNcoF | TGATAACCATGGAAGGTAAAATAATTGGTATCGACCTGG |
| DnaKBamHR | CGATTAGGATCCTCATTATTATTTTTTGTCTTTGACTTCTTCAAATTC |
| GrpENdeF | AGCTGACATATGAGTAGTAAAGAACAGAAAACGCC |
| GrpEXhoR | CGATTACTCGAGTCATTATTAAGCTTTTGCTTTCGCTACAG |

**Supplementary Table S3.** Predicted DnaK binding sequences. The predicted DnaK binding sequences were analyzed using Limbo algorithm with the best overall prediction option and threshold score of 11.08.

| POI | Binding area | Binding motif | Score |
|---|---|---|---|
| ScFv (249 aa, 2 disulfide bonds) | 35-41 | MNWIRQT | 11.3 |
| | 77-83 | KNTLYLQ | 12.2 |
| | 79-85 | TLYLQMT | 16.8 |
| | 106-112 | DFFDYWG | 11.7 |
| | 166-172 | SNYLAWY | 22.8 |
| | 181-187 | QLLIYYA | 19.5 |
| HIV1-Pr (106 aa, 1 disulfide bond) | Not found | Not found | Not found |
| BR2ScFv (267 aa, 2 disulfide bonds) | 10-16 | GRLLRRL | 12.3 |
| | 11-17 | RLLRRLL | 12.3 |
| | 12-18 | LLRRLLR | 12.7 |
| | 13-19 | LRRLLRG | 12.3 |
| | 54-60 | MNWIRQT | 11.3 |
| | 96-102 | KNTLYLQ | 12.2 |
| | 98-104 | TLYLQMT | 16.8 |
| | 125-131 | DFFDYWG | 11.7 |
| | 184-190 | SNYLAWY | 22.8 |
| | 199-205 | QLLIYYA | 19.5 |
| UGD (394 aa, no disulfide bond) | 16-22 | GLLIAQN | 12.7 |
| | 75-81 | DYVIIAT | 11.2 |
| | 258-264 | TKQLLAN | 12.6 |

| | | | |
|---|---|---|---|
| | 300-306 | GIYRLIM | 11.9 |
| | 301-307 | IYRLIMK | 13.5 |
| | 302-308 | YRLIMKS | 16 |
| Adh1p (354 aa, no disulfide bond) | 33-39 | ELLINVK | 13 |
| | 78-84 | ENVKGWK | 13.9 |
| | 217-223 | EVFIDFT | 13.5 |
| | 257-263 | TRYVRAN | 12.5 |
| UbiC (208 aa, no disulfide bond) | 22-28 | TFLRYNA | 12.4 |
| | 63-69 | LDWLLLE | 11.7 |
| | 65-71 | WLLLEDS | 13.9 |
| | 110-116 | RYWLREI | 11.1 |
| | 153-159 | GRYLFTS | 14.7 |
| | 189-195 | LLLTELF | 13.1 |
| | 193-199 | ELFLPAS | 15.5 |
| Leptin (152 aa, 1 disulfide bond) | 14-20 | IKTIVTR | 11.3 |
| | 16-22 | TIVTRIN | 11.2 |
| | 63-69 | QILTSMP | 11.9 |
| | 95-101 | SCHLPWA | 12.4 |
| BMP2 (121 aa, 3 disulfide bond) | 90-96 | MLYLDEN | 11.3 |
| | 97-103 | EKVVLKN | 11.5 |
| | 98-104 | KVVLKNY | 11.9 |
| N-terminal sfGFP (217 aa, no disulfide bond) | 40-46 | GKLTLKF | 12.4 |
| | 41-47 | KLTLKFI | 12.8 |
| | 52-58 | KLPVPWP | 13 |
| | 198-204 | NHYLSTQ | 11.1 |

**Supplementary Figure S1**

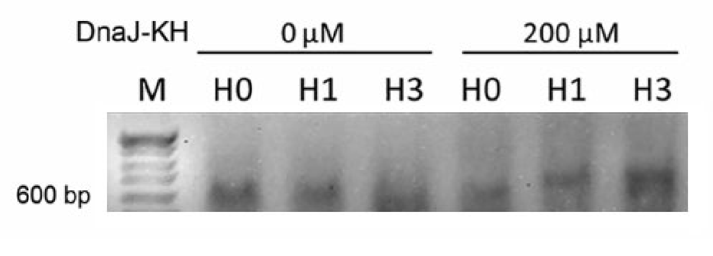


**Figure S1. Gel retardation assay to confirm the binding of the DnaJ-KH to the binding hairpins in the CRAS system**. Shifted migration of HIV-1 protease mRNA with 1 (H1) and three 3′UTR KH hairpins (H3) observed in the presence of 200 μM purified DnaJ-KH.

**Supplementary Figure S2**

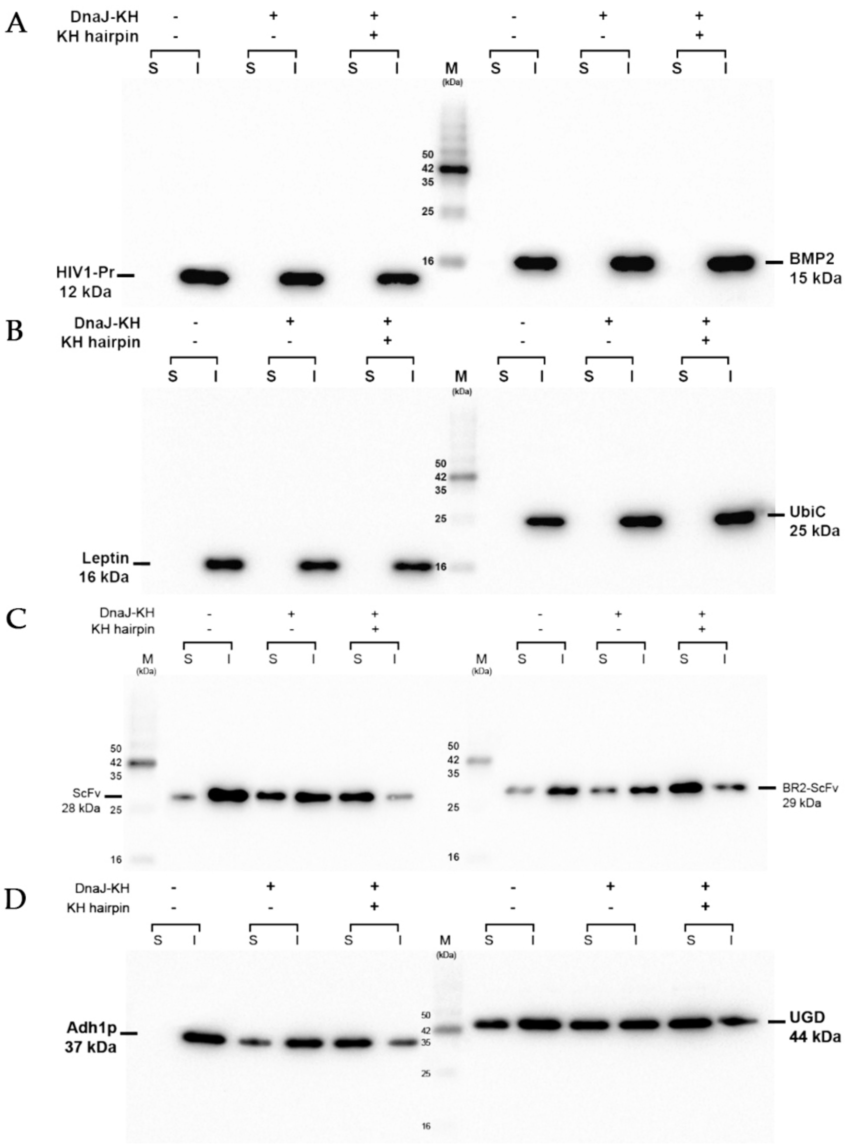


**Figure S2. Western Blot analysis of selected recombinant proteins expressed by CRAS system**. Lanes S, soluble fraction; Lanes I, insoluble fraction; Lanes M, West-View 10 kDa Western marker (ELPIS Biotech). (A) The expression of HIV1-Pr (left) and BMP2 (right); (B) The expression of Leptin (left) and UbiC (right); (C) The expression of ScFv (left) and BR2-ScFv (right); (D) The expression of Adh1p (left) and UGD (right). Bands corresponding each recombinant proteins are indicated on the sides of the blot. For the evaluation of *in vivo* solubilization effect of CRAS system, the DnaJ-KH without His tag was used.

**Supplementary Figure S3**

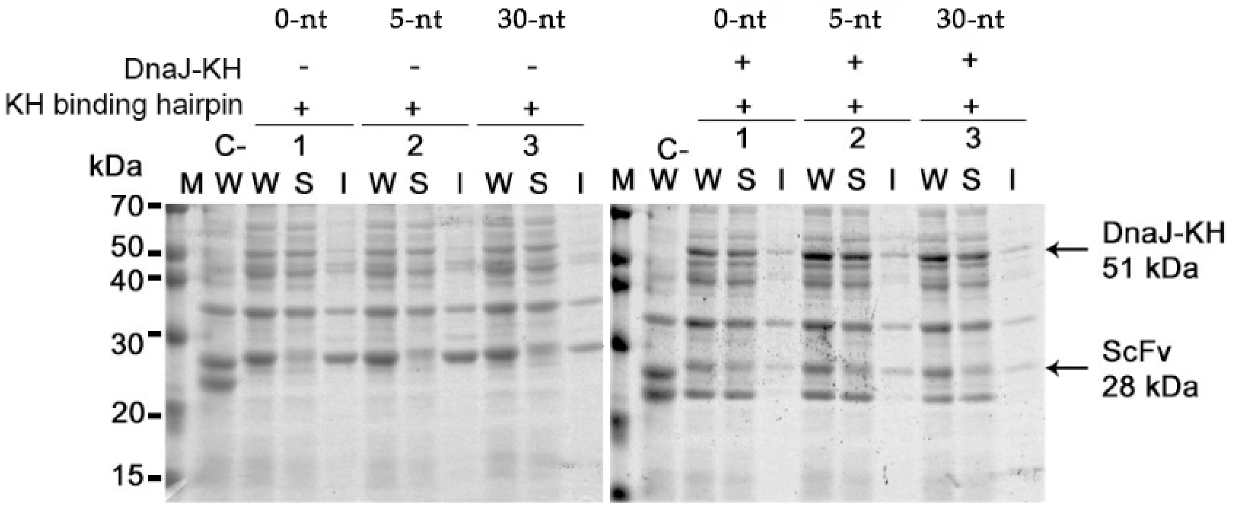


**Figure S3. Effect of spacer length between the stop codon and the 3′UTR binding loop on the efficacy of the CRAS system**. Coomassie blue stained 10% SDS-PAGE results demonstrating the efficacy of the CRAS system on improving the solubilization of the ScFv in BL21(DE3) strain after 4 h of induction using 0.5 M IPTG. The distance between the stop codon of *scfv* and KH binding loop is indicated on top of each group. Lanes 1, no spacer (0-nt) between the stop codon and 3′UTR binding loop; Lanes 2, in the presence of 5-nt spacer between the stop codon and 3′UTR binding loop; Lanes 3, in the presence of 30-nt spacer between the stop codon and 3′UTR binding loop. Lanes M, Mid-range range pre-stained marker (ELPIS Biotech); Lanes W, whole cell lysate fraction; Lanes S, soluble fraction; Lanes I, insoluble fraction. Bands corresponding to DnaJ-KH (51 kDa) and ScFv (28 kDa) are indicated by arrows.

**Supplementary Figure S4**

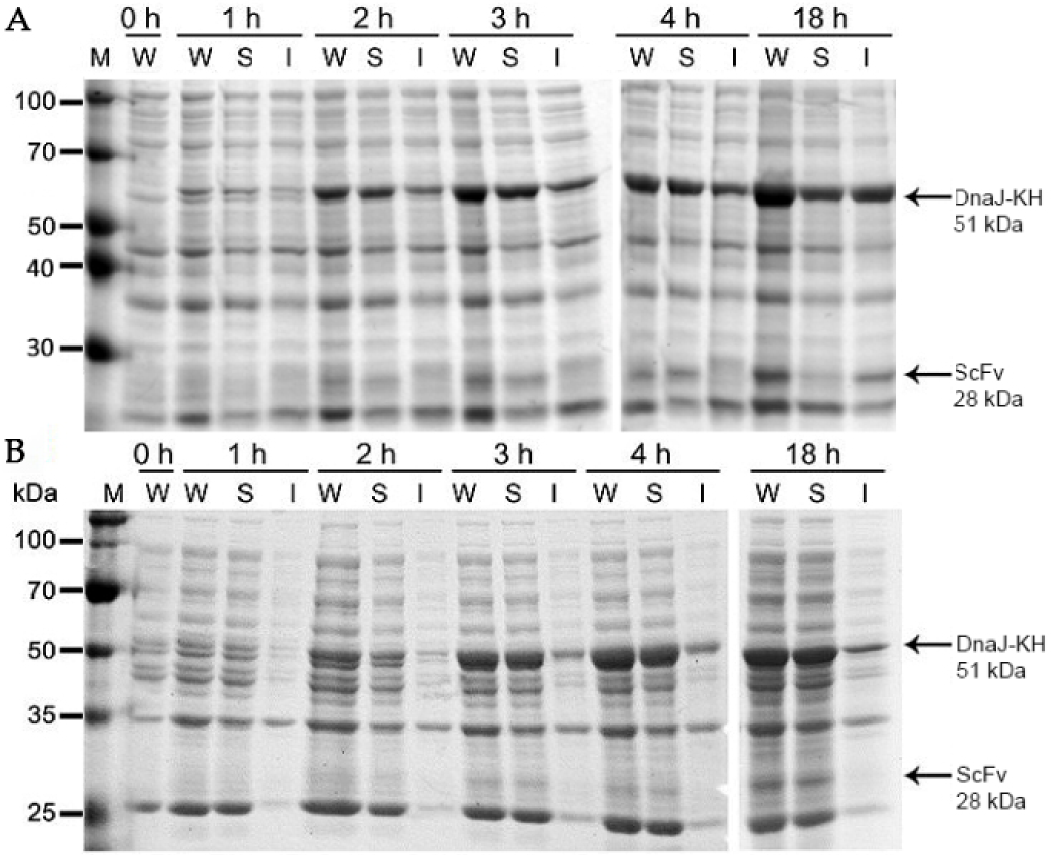


**Figure S4. Time course solubilization of ScFv in *E. coli* BL21(DE3).** (A) Time course solubilization of ScFv using the CRAS system with 1-loop and (B) 3-loop design; Lane M, Mid-range range pre-stained marker (ELPIS Biotech); Lanes W, whole cell lysate fraction; Lanes S, soluble fraction; Lanes I, insoluble fraction. The time shown on top is the period of cell incubation after the IPTG induction. Bands corresponding to DnaJ-KH (51 kDa) and ScFv (28 kDa) are indicated by arrows.

**Supplementary Figure S5**

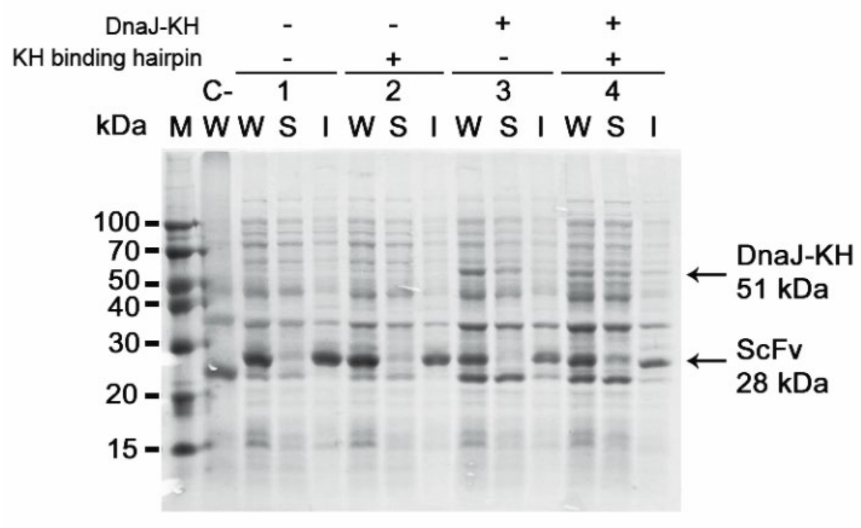


**Figure S5. Efficacy of the CRAS system on improving the solubilization of ScFv in the *dnaK* knockout BL21(DE3) strain after 4 h of induction.** Lane C-, whole cell lysate of *E. coli* BL21(DE3) harbouring pET16b and pAMT7 (negative control); Lanes 1–4, expression pattern of ScFv in the absence of binding loop and DnaJ-KH (Lanes 1); in the presence of binding loop and the absence of DnaJ-KH (Lanes 2); in the absence of binding loop and the presence of DnaJ-KH (Lanes 3); and in the presence of binding loop and DnaJ-KH (CRAS system) (Lanes 4). Lane M, Mid-range pre-stained marker (ELPIS Biotech); Lanes W, whole cell lysate fraction; Lanes S, soluble fraction; Lanes I, insoluble fraction. Bands corresponding to DnaJ-KH (51 kDa) and ScFv (28 kDa) are indicated by arrows.

**Supplementary Figure S6**

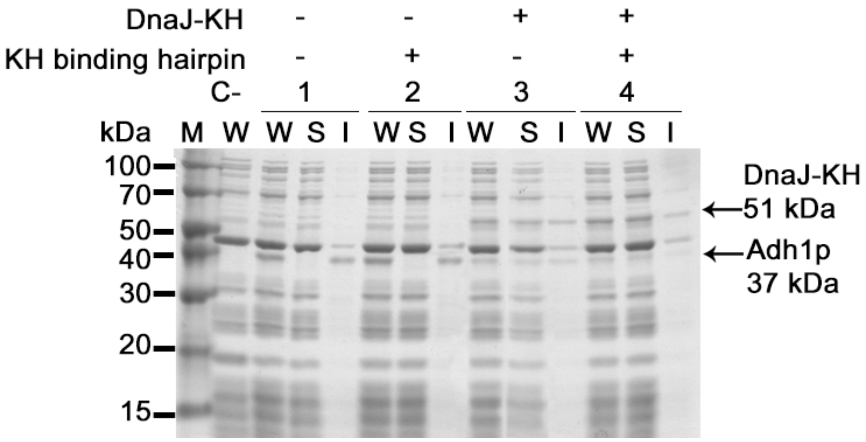


**Figure S6.** ***in vitro*** **solubilization of Adh1p with DnaJ-KH. pET16b-Adh and pAMT7.** Lane (C-); pET16b-Adh and pAMT7 (1); pET16b-Adh3L and pAMT7 (2); pET16b-Adh and pAMT7-DnaJ-KH (3); and pET16b-Adh3L and pAMT7-DnaJ-KH (4) were used as templates for *in vitro* translation using the PURExpress® In Vitro Protein Synthesis Kit. Samples were collected after 4 h of incubation at 37°C and examined by 10% SDS-PAGE.

**Supplementary Figure S7**

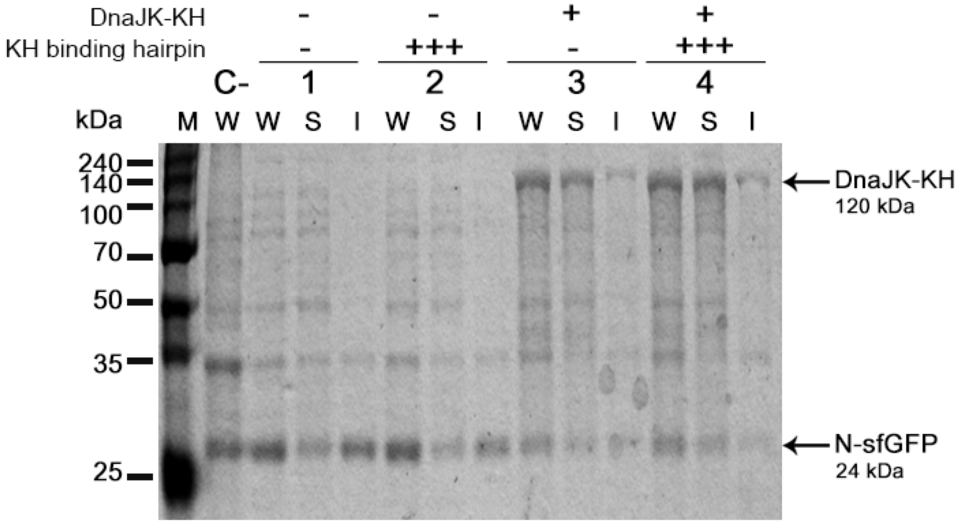


**Figure S7. Efficacy of the CRAS system on improving the solubilization of N-terminal GFP (N-sfGFP) in *E. coli* BL21(DE3) after 4 h of induction.** Lane C-, whole cell lysate of *E. coli* BL21(DE3) harbouring pET16b and pAMT7 (negative control); Lanes 1–4, expression pattern of N-sfGFP in the absence of binding loop and DnaJK-KH (Lanes 1); in the presence of binding loop and the absence of DnaJK-KH (Lanes 2); in the absence of binding loop and the presence of DnaJK-KH (Lanes 3); and in the presence of 3 repeats of binding loops and DnaJK-KH (CRAS system) (Lanes 4). Lane M, Broad

range pre-stained marker (ELPIS Biotech); Lanes W, whole cell lysate fraction; Lanes S, soluble fraction; Lanes I, insoluble fraction. Bands corresponding to DnaJK-KH (120 kDa), and N-sfGFP (24 kDa) are indicated by arrows. The repeat number of binding loops is indicated by the number of plus symbols.

**Supplementary Figure S8**

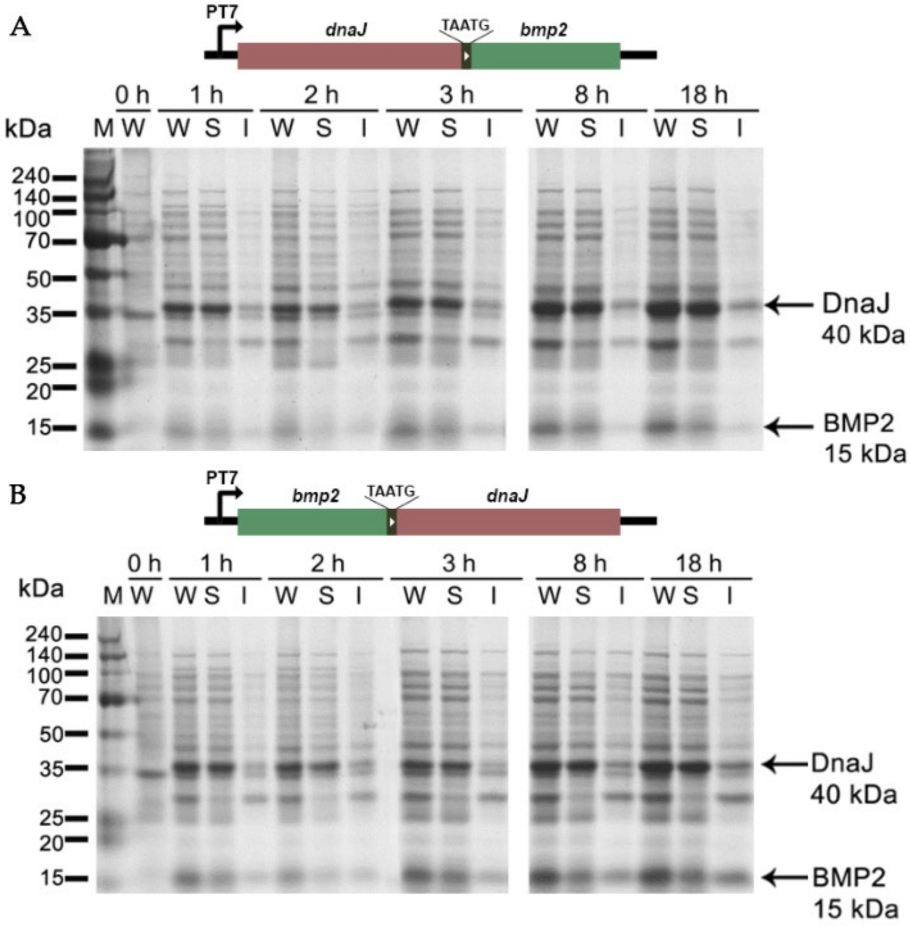


**Figure S8. Time course solubilization of BMP2 in the application of the CLEX system.** (A) Expression pattern of BMP2 in the CLEX system when placed as the second cistron and DnaJ as the first cistron, and (B) the reverse arrangement. Lane M, Broad range pre-stained marker (ELPIS Biotech); W: Whole cell lysate; S: Soluble fraction; I: Insoluble fraction. Bands corresponding to DnaJ (40 kDa) and BMP2 (15 kDa) are indicated by arrows.

**Supplementary Figure S9**

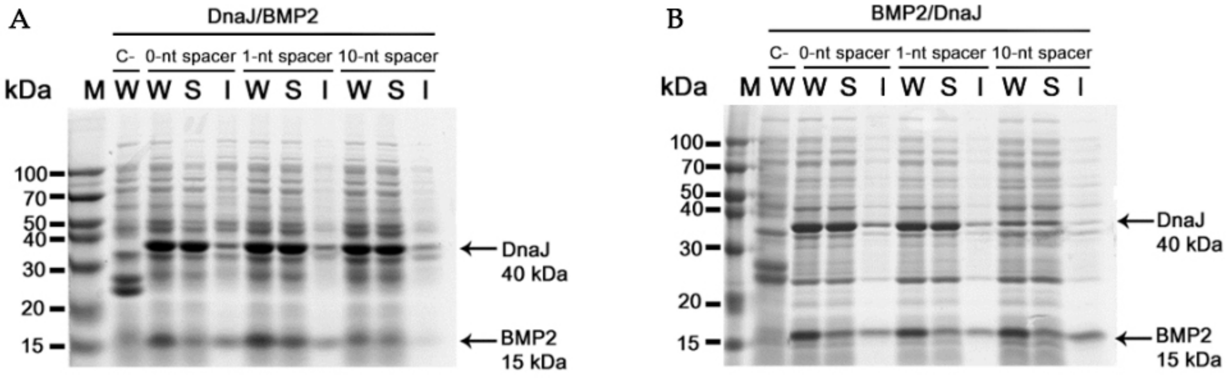


**Figure S9. Effect of spacer length between the stop codon of the first cistron and start codon of the second cistron on the efficacy of the CLEX system.** Coomassie blue-stained 10% SDS-PAGE demonstrating the efficacy of the CLEX system with 0-, 1-, and 10-nt spacer between (A) stop codon of DnaJ (first cistron) and start codon of BMP2 (second cistron), (B) stop codon of DnaJ (first cistron) and start codon of BMP2 (second cistron) on improving the solubilization of BMP2 in the BL21(DE3) strain after 4 h induction using IPTG. Lane C-, Whole cell lysate of *E. coli* BL21(DE3) harbouring pET16b and pAMT7 (negative control); Lane M, Mid-range pre-stained marker (ELPIS Biotech); W: Whole cell lysate; S: Soluble fraction; I: Insoluble fraction. Bands corresponding to DnaJ (40 kDa) and BMP2 (15 kDa) are indicated by arrows.

**Supplementary Figure S10**

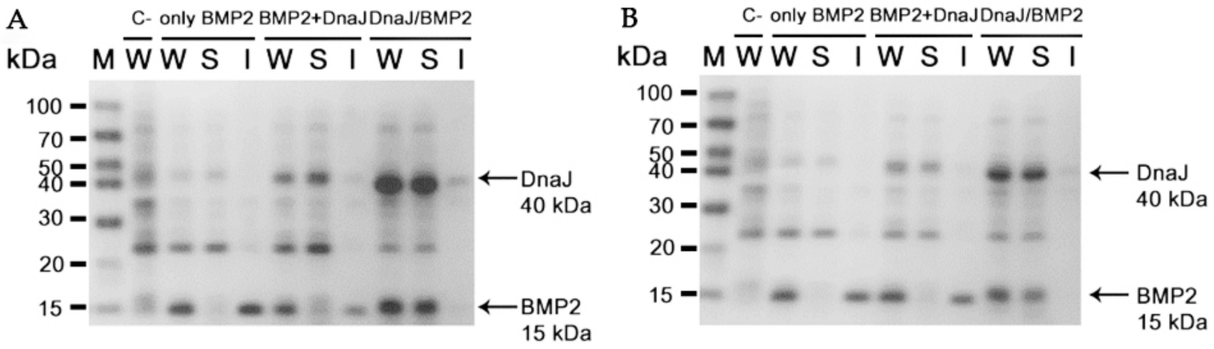


**Figure S10. Efficacy of the CLEX system on improving the solubilization of BMP2 in (A) *dnaJ* and (B) *dnaK* knockout BL21(DE3) strain after 4 h of induction.** Lane C-, whole cell lysate of *E. coli* BL21(DE3) harbouring pET16b and pAMT7 (negative control); “only BMP2”, only BMP2 is overexpressed; “BMP2+DnaJ”, BMP2 is co-expressed with DnaJ; “DnaJ/BMP2”, BMP2 and DnaJ are expressed in CLEX system when DnaJ as the first cistron and BMP2 as the second cistron. Lane M, Mid-range pre-stained marker (ELPIS Biotech); Lanes W, whole cell lysate fraction; Lanes S, soluble fraction; Lanes I, insoluble fraction. Bands corresponding to DnaJ (40 kDa), and BMP2 (15 kDa) are indicated by arrows.

**Supplementary Figure S11**

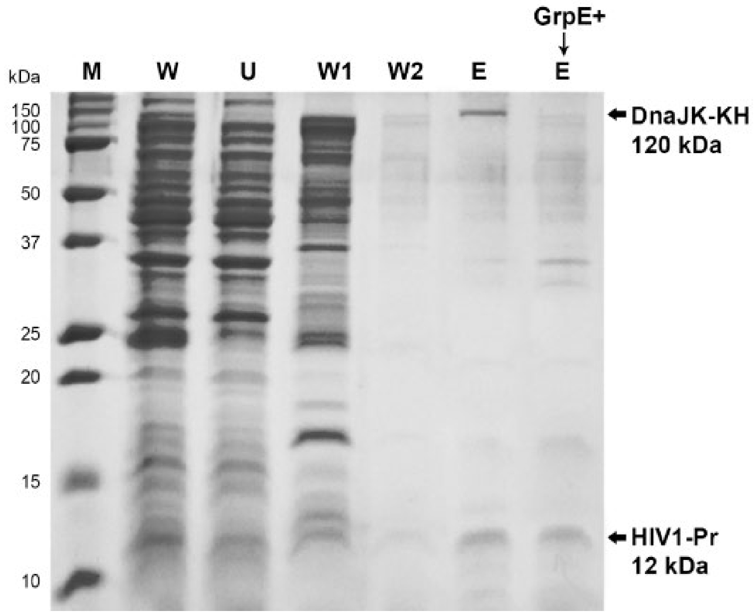


**Figure S11. DnaJK-KH is co-purified with HIV-1 protease via Ni-IDA resin.** Coomassie blue-stained 10% SDS-PAGE showing the purification of HIV1-Pr (with His tag and 3 x 3′UTR KH binding domains) using the CRAS system. Lane M, Precision Plus Protein Dual Xtra Prestained Standards (Bio-Rad); W: whole cell lysate; U: unbound fraction; W1 and W2: washing fractions using 5 mM and 60 mM imidazole, respectively; E: elution fractions using 500 mM imidazole. The second E lane with the GrpE+ is the elution fraction of the sample co-expressing GrpE with CRAS system. Bands corresponding to DnaJK-KH (120 kDa) and HIV1-Pr (12 kDa) are indicated by arrows.

**Supplementary Figure S12**

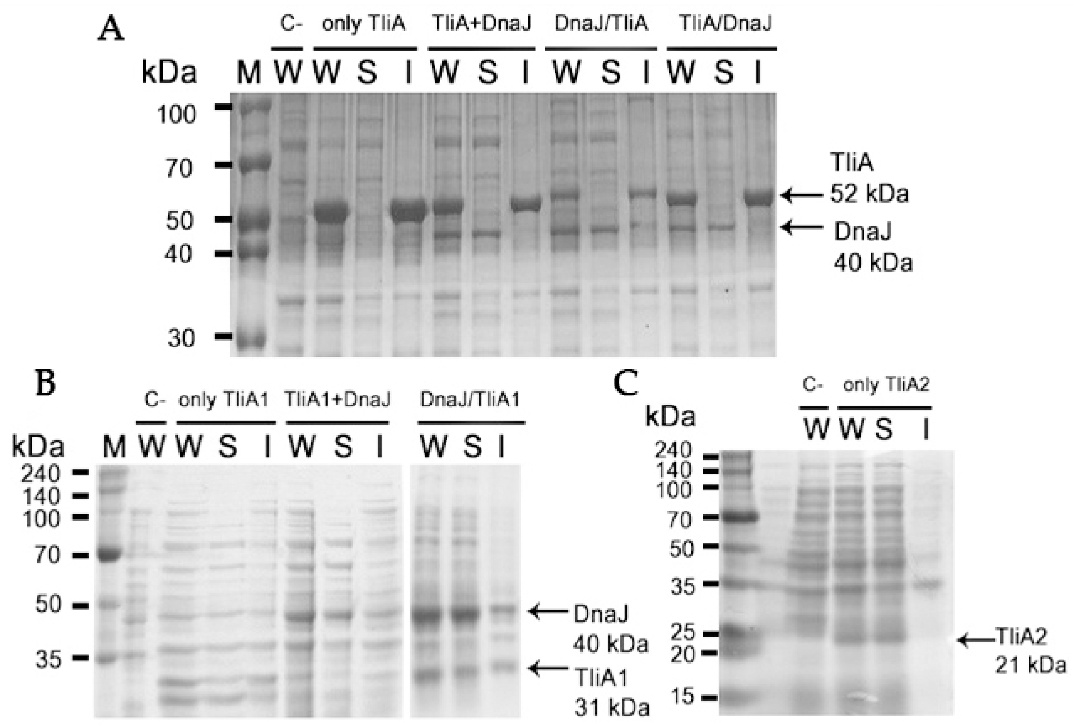


**Figure S12. Effect of the CLEX system on the solubilization** of (A) TliA lipase (TliA) and (B) TliA fragment containing amino acids 1–300 (TliA1), and the expression pattern of (C) TliA fragment containing amino acids 301–493 (TliA2) *in E. coli* BL21(DE3) after 4 h induction using IPTG. Lane C-, whole cell lysate of *E. coli* BL21(DE3) harbouring pET16b and pAMT7 (negative control); "only TliA, TliA1, or TliA2", expression pattern of recombinant proteins in the absence of DnaJ; "TliA+DnaJ or TliA1+DnaJ", expression pattern of recombinant proteins in the presence of DnaJ; "DnaJ/TliA or DnaJ/TliA1", expression pattern of TliA and TliA1 in the CLEX system when placed as the second cistron, respectively, with DnaJ as the first cistron; "TliA/DnaJ", DnaJ placed as the second cistron. Lane M, Mid-range or broad range pre-stained marker (ELPIS Biotech); Lanes W, whole cell lysate fraction; Lanes S, soluble fraction; Lanes I, insoluble fraction. Bands corresponding to DnaJ (40 kDa), TliA (52 kDa), TliA1 (31 kDa), and TliA2 (21 kDa) are indicated by arrows.
